# Phenotypic characterization with somatic genome editing and gene transfer reveals the diverse oncogenicity of ependymoma fusion genes

**DOI:** 10.1186/s40478-020-01080-8

**Published:** 2020-11-23

**Authors:** Mutsumi Takadera, Kaishi Satomi, Frank Szulzewsky, Patrick J. Cimino, Eric C. Holland, Tetsuya Yamamoto, Koichi Ichimura, Tatsuya Ozawa

**Affiliations:** 1 Division of Brain Tumor Translational Research, National Cancer Center Research Institute, 5-1-1 Tsukiji, Chuo-ku, Tokyo, 104-0045 Japan; 2 Department of Neurosurgery, Yokohama City University, Yokohama, 236-0027 Japan; 3 Department of Diagnostic Pathology, National Cancer Center Hospital, Tokyo, 104-0045 Japan; 4 Human Biology Division, Fred Hutchinson Cancer Research Center, 1100 Fairview Avenue North, Mailstop C3-168, Seattle, WA 98109 USA; 5 Department of Laboratory Medicine and Pathology, University of Washington, Seattle, WA 98104 USA; 6 Seattle Tumor Translational Research Center, Fred Hutchinson Cancer Research Center, 1100 Fairview Avenue North, Seattle, WA 98109 USA

**Keywords:** Supratentorial ependymoma, RELA, CRISPR/Cas9 system, Brain tumor mouse model, Gene rearrangement

## Abstract

Recurrent *RELA* and *YAP1* fusions are intimately associated with tumorigenesis in supratentorial ependymomas. Chromothripsis and focal copy number alterations involving 11q are hallmarks of these tumors. However, it is unknown whether the chromosomal alterations are a direct causal event resulting in fusion transcripts. In addition, the biological significance of the *RELA* fusion variants and Y*AP1* fusions is not yet fully characterized. In this study, we generated gene rearrangements on 11q with the CRISPR/Cas9 system and investigated the formation of oncogenic ependymoma fusion genes. Further, we examined the oncogenic potential of *RELA* fusion variants and *YAP1* fusions in a lentiviral gene transfer model. We observed that endogenous *RELA* fusion events were successfully induced by CRISPR/Cas9-mediated genome rearrangement in cultured cells. In vivo genome editing in mouse brain resulted in the development of ependymoma-like brain tumors that harbored the *Rela* fusion gene. All *RELA* fusion variants tested, except a variant lacking the Rel homology domain, were able to induce tumor formation, albeit with different efficacy. Furthermore, expression of *YAP1*-*FAM118B* and *YAP1*-*MAMLD1* fusions induced the formation of spindle-cell-like tumors at varying efficacy. Our results indicate that chromosomal rearrangements involving the *Rela* locus are the causal event for the formation of *Rela* fusion-driven ependymomas in mice. Furthermore, the type of *RELA.* fusion might affect the aggressiveness of tumors and that the Rel homology domain is essential for the oncogenic functions of *RELA.* fusions. The *YAP1* fusion genes are also oncogenic when expressed in mice.

**Electronic supplementary material:**

The online version of this article (10.1186/s40478-020-01080-8) contains supplementary material, which is available to authorized users.

## Introduction

Ependymomas are primary tumors of the central nervous system which occur throughout the craniospinal axis. These tumors occur in all age groups, but intracranial tumors are more common in children [16, 20]. The current best available treatment for these patients is radical surgical removal, followed by local radiation therapy [18]. Since there is currently no effective chemotherapy for ependymomas, recurrent tumors may be lethal in some cases if not fully surgically resected [2, 9, 14]. Therefore, survival of ependymoma patients has not been drastically improved over the past decades [2], indicating that the development of a new treatment regimen is necessary for these intractable tumors.

Recent large-scale genomic studies revealed that ependymomas segregate into distinct molecular subgroups, which are strongly associated with anatomical location [10, 20, 21]. Supratentorial ependymomas are classified into subependymoma (ST-SE) and ependymoma (ST-EPN) subgroups. The ST-EPN subgroup can be further stratified by mutually exclusive recurrent *RELA* and *YAP1*-related gene fusions. These ST-EPN gene fusions not only have diagnostic utility, but also provide potential therapeutic insights into these fusion-positive tumors.

The ST-EPN-RELA subgroup accounts for the majority of supratentorial ependymomas and is characterized by the presence of a gene fusion between *C11orf95* and *RELA* gene [20, 21]. The *C11orf95*-*RELA* fusion (*RELA*^FUS^) genes are caused by chromothripsis, a single catastrophic genomic rearrangement involving 11q, consequently resulting in several *RELA*^*FUS*^ variants [21]. *RELA*^FUS1^ (Type 1) and *RELA*^FUS2^ (Type 2), the two most frequent variants, were able to induce human ependymoma-like tumors when expressed in murine neural stem cells, using either the RCAS/tv-a system for retroviral gene transfer or an allograft model [17, 21]. Although these findings strongly suggest that *RELA*^FUS^ genes are responsible for human ependymoma formation, the oncogenic potential of other *RELA*^FUS^ variants has not been determined yet. In addition, several other types of fusion transcripts involving genes on 11q were also identified concomitant with the *RELA*^FUS^ transcripts in supratentorial ependymomas [21]. This catastrophic genomic event might affect the status of other genes on 11q, and thereby contribute to the tumorigenesis of *RELA*^FUS^-positive tumors. However, the influence of these complex genomic rearrangements has not been captured in previous mouse models [17, 21].

The ST-EPN-YAP1 subgroup is a less common type of supratentorial ependymoma and harbors *YAP1*-related fusions, found as *YAP1*-*MAMLD1* and *YAP1*-*FAM118B* gene fusions [20]. In contrast to the ST-EPN-RELA subgroup, the genome of these tumors is relatively stable except for a focal copy number alteration involving the *YAP1* gene locus on 11q [20], thus suggesting that a more limited number of gene alterations may be sufficient to drive the formation of ST-EPN-YAP1 tumors. The oncogenic potential of YAP1-MAMLD1 has recently been demonstrated by the forced expression via in utero gene transfer in the embryonic subventricular zone [19]. However, the *YAP1*-*FAM118B* fusion failed to induce the formation of brain tumors in the same study. On the other hand, the tumor-forming potential of both fusions was observed when expressed in neonatal brains using the RCAS/tv-a system [23], thus suggesting a potentially different tumorigenic mechanism between these *YAP1* fusion genes.

To clarify the biological significance of different chromosomal alterations in supratentorial ependymoma formation, we examined the oncogenic potential of ependymoma gene fusions in two independent mouse tumor models. First, we directly engineered chromosomal rearrangements in mice using the CRISPR/Cas9 system and reproduced the formation of oncogenic gene fusions found in human ependymoma. Second, we demonstrated the oncogenic potential of several variants of *RELA*^*FUS*^ and *YAP1*-related fusion genes using in vivo lentiviral gene transfer into the brain of young adult and/or neonatal mice.

## Materials and methods

### Cell culture

293T cells (ATCC #CRL-3216) and NIH3T3 mouse fibroblasts (ATCC #CRL-1658) were obtained from ATCC and maintained with minor modifications according to the manufacture’s protocol. The primary mouse embryonic fibroblasts were established from *Nestin*-*Cre*^+*/*−^;*Cag*-*Cas9*^+*/*+^ mice and maintained in Dulbecco’s modified Eagle’s media (Gibco) supplemented with 10% fetal bovine serum (Gibco). Mouse neurospheres were generated and maintained as previously described [17].

### Mouse

*Nestin*-*Cre* and *Cag*-*Cas9* mice were purchased from The Jackson Laboratories (Stock No. 003771 and 027650) and crossed to obtain *Nestin*-*Cre*^+*/*−^*;Cag*-*Cas9*^+*/*+^ mice. Genotyping protocol was described in the Additional file 1: Supplementary Methods [3].

### Plasmids and lentivirus production

The pTomo lentiviral vector system was used as previously described [12]. LV-EDIT-mRela^fus^ vector was generated by Gibson Assembly of relevant U6-sgRNA fragments into the pTomo vector [26]. The sgRNA sequences targeting *Cdkn2a* and control were previously described [1, 27]. Other sgRNAs were designed using the CRISPRDirect tool [15]. The details of vector constructs and lentivirus production are described in the Additional file 1: Supplementary Methods. All vectors and sgRNA sequences used in this study are listed in Additional file 2 and 3: Table S1 and S2.

### Generation of mouse brain tumors

Mice were injected with the relevant lentivirus and were monitored until they developed symptoms of disease or, if symptom-free, until 5 months after the lentivirus injection. Kaplan–Meier analysis demonstrating symptom-free survival of mouse brain tumors was performed using log-rank test in the GraphPad Prism 8 software. A value of *p* < 0.05 was considered significant in this study. See Additional file 1: Supplementary Methods for details.

### RT-PCR

Total RNA was extracted from cultured cells, flash-frozen brain tumor tissues, or formalin-fixed paraffin-embedded (FFPE) tissues using a miRNeasy Mini or RNeasy FFPE kit (Qiagen) according to the manufacturer’s protocol. cDNA libraries were synthesized with the Superscript IV VILO Master Mix (Invitrogen) according to the manufacturer’s protocol and used for subsequent PCR amplification. PCR reaction was performed in a 45-μl reaction volume under the following conditions: 0.3 μl of AmpliTaq Gold360 DNA polymerase (AppliedBiosystems), 4.5 μl of AmpliTaq Gold 360 buffer, 9 μl of GC enhancer, 1.8 μl of 25 mM Magnesium chloride, 1.2 μl of 10 mM dNTP mix, primer pairs (0.5 μM each), 50 ng of the cDNA template and autoclaved distilled water up to 45 μl. The protocol included pre-denaturation for 10 min at 95 °C, denaturation for 30 s at 95 °C, annealing for 30 s at 55 °C, extension for 30 s at 72 °C for 40 cycles and final extension for 10 min at 72 °C. The PCR products were resolved by electrophoresis on 2% agarose gel with GelRed Nucleic Acid Gel Stain (Biotium). Primer sequences are listed in Additional file 4: Table S3.

### Western blot analysis

Cells were cultured, lysed, and processed for western blotting by standard methods as previously described [17]. α-tubulin was used as an internal normalization control. Antibodies used in this study are listed in Additional file 5: Table S4.

### H&E staining and immunohistochemistry

Mouse brains were paraffin-embedded, sectioned, and stained with hematoxylin and eosin (H&E) and immunohistochemistry (IHC) by standard methods (See Additional file 1: Supplementary Methods for details). In all H&E and IHC photomicrographs, dashed boxes in the top panels denote the enlarged regions as shown in the bottom panels. The length of all scale bars is 100 μm.

## Results

### Lentiviral delivery of *C11orf95*-*RELA* type 1 fusion into mouse neural stem cells induces human ependymoma-like tumors

To examine the oncogenic potential of fusion genes found in human ependymomas, we initially established a model system for in vivo lentiviral gene transfer using the CRISPR/Cas9 system [7, 12]. Given previous studies where expression of the *C11orf95*-*RELA* type 1 fusion (*RELA*^FUS1^) was sufficient to induce formation of ependymoma-like brain tumors in mice [17, 21], we generated a pTomo-*RELA*^FUS1^-*HA* (containing a C-terminal human influenza hemagglutinin [HA] tag) lentiviral vector which can induce the exogenous gene expression in a cell-type-specific manner using the Cre-loxP system (Additional file 6: Fig. S1A and B) [12]. Since loss of *CDKN2A* is a common event in the ST-EPN-RELA subgroup [20, 21], we also examined the impact of *Cdkn2a* inactivation on tumorigenesis by inserting sgRNAs against *Cdkn2a* or a non-targeting control sequence into the lentiviral vector (Additional file 6: Fig. S1A and B). These constructs were then tested in *Nestin*-*Cre*^+*/*−^*;Cag*-*Cas9*^+*/*+^ mice to target Nestin^+^ radial glia, the putative cell of origin for ependymomas (Additional file 6: Fig. S1C and D) [24].

**Fig. 1:**
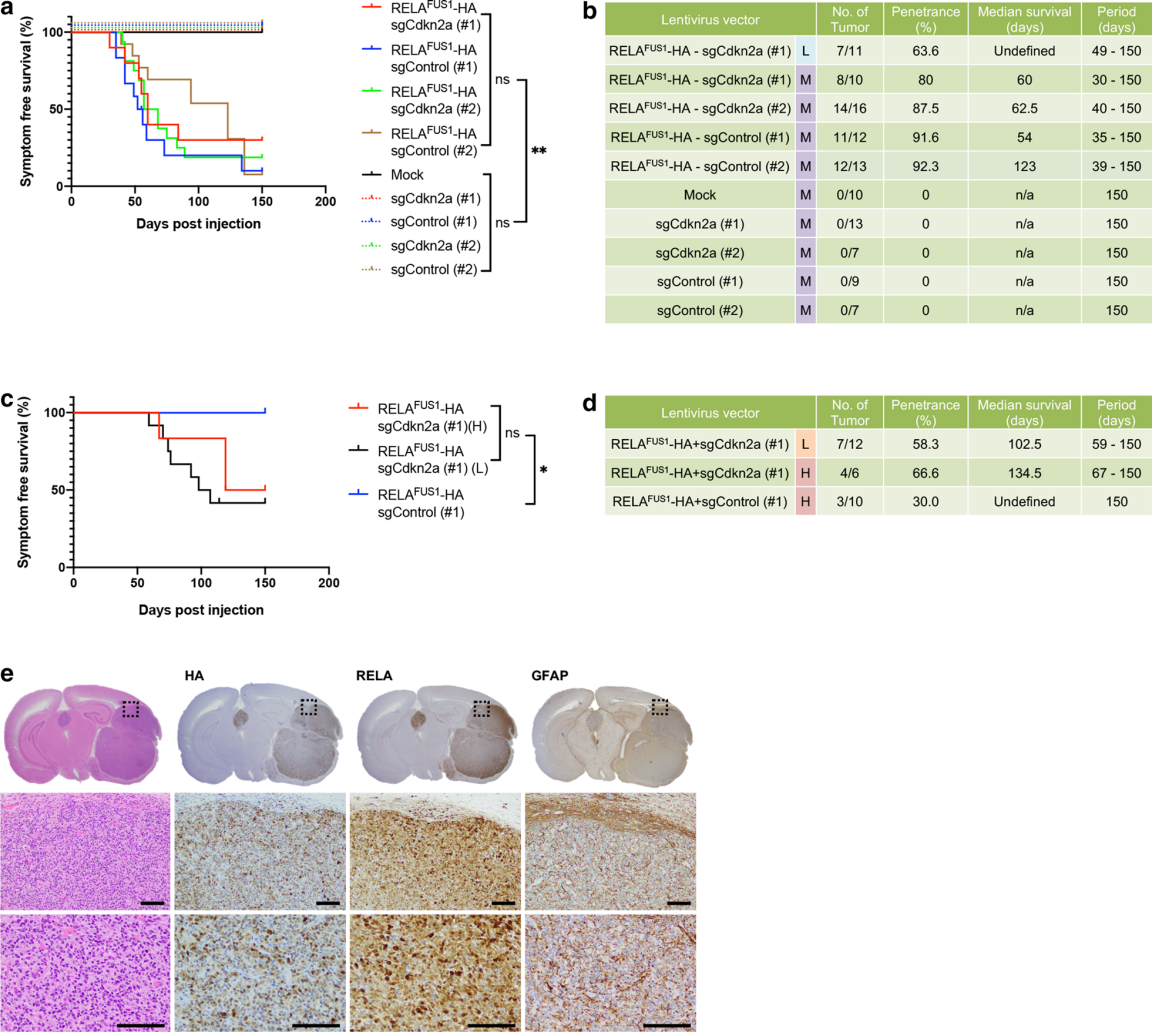
Lentiviral gene transfer of *C11orf95*-*RELA* type 1 fusion into mouse neural stem cells induces human ependymoma-like tumors. **a** Kaplan–Meier survival curves of the pTomo-RELA^FUS1^-HA-induced brain tumors. Lentiviruses were injected into neonatal pups brain in *Nestin*-*Cre*^+*/*−^*;Cag*-*Cas9*^+*/*+^ mice. Two different target sequences (#1 and #2) were tested for each sgRNA (n.s., not significant, **p < 0.01). **b** Summary of pTomo-RELA^FUS1^-HA lentivirus injections. L, lower titer; M, medium titer; n/a, not applicable (**c**, **d**) Kaplan–Meier survival curves and Summary of the pTomo-RELA^FUS1^-HA-induced brain tumors. Lentiviruses were injected into brain in 3–5 weeks-old *Nestin*-*Cre*^+*/*−^*;Cag*-*Cas9*^+*/*+^ mice (n.s., not significant, *p < 0.05. L, lower titer; H, higher titer). **e** Representative H&E and IHC stained slides for HA tag, RELA and GFAP antibodies in the RELA^FUS1^-HA + sgControl (#1)-induced brain tumor in *Nestin*-*Cre*^+*/*−^*;Cag*-*Cas9*^+*/*+^ mice (n = 3)

Injection of the lentivirus encoding for *RELA*^FUS1^-sgCdkn2a into the brain of neonatal pups, brain tumor formation was observed around 2 months post-injection (Fig. 1a). Interestingly, the overall tumor latency was considerably shorter compared to our previous model (Fig. 1a, b) [17]. We did not observe a significant difference in tumor incidence or latency between experimental groups with and without additional *Cdkn2a* loss (sg*Cdkn2a* versus sgControl) (Fig. 1a, b and Additional file 6: Fig. S1E). This finding is consistent with our previous study in which *Cdkn2a* loss had no impact on the *RELA*^FUS1^-induced brain tumor formation [17]. We did not observe tumor formation in control groups injected with an empty vector or lentiviruses containing sg*Cdkn2a* or sgControl only (Fig. 1a, b).

In addition to pediatric cases, ST-EPN-RELA tumors also occur in adults [16, 20]. Thus, to examine whether expression of RELA^FUS^ is able to induce tumor formation in the adult population, we injected our lentivirus encoding for *RELA*^FUS1^ into the subventricular zone of *Nestin*-*Cre*^+*/*−^*;Cag*-*Cas9*^+*/*+^ young adult mice. We found that brain tumors were reproducibly formed in the adult mice, albeit with a longer latency compared to our injections in neonatal mice (Fig. 1a–d). In contrast to neonatal pup injection, we observed a significant impact of additional *Cdkn2a* deletion in adult mice, suggesting different susceptibility to this gene inactivation between neonatal and adult brains (Fig. 1a–d).

Mouse brain tumors typically presented as well-circumscribed and uniform highly cellular masses adjacent to the ventricular system, or occasionally in the brain parenchyma (Fig. 1e and Additional file 6: Fig. S1F). The tumors were composed of relatively monomorphic cells with oval nuclei containing finely granular chromatin. Branching capillaries characteristic for human ST-EPN-RELA ependymomas were commonly seen throughout the mouse tumors [6, 17, 21]. Clear-cell change, another characteristic histological finding in human ST-EPN-RELA ependymomas, were occasionally observed. Ependymal rosettes, ependymal canals, and perivascular pseudorosettes were rarely seen in these tumors. Occasionally, the tumors demonstrated a fascicular architectural growth pattern, with spindled cells densely interwoven with epithelioid-type cells, reminiscent of high-grade glioma or sarcoma. Immunostaining for RELA and HA-tag antibodies displayed predominant nuclear immunopositivity, indicating successful vector expression in the tumors. Similar to human ependymomas, there was consistent extensive immunopositivity for the glial marker GFAP detected in the mouse tumor cells (Fig. 1e and Additional file 6: Fig. S1F). No pathological lesions were found in control mice (mock injection or injection of sg*Cdkn2a* or sgControl alone) at the study endpoint.

Taken together, these *RELA*^FUS1^ tumors showed similar biological and histological features to previous *RELA*^FUS1^ models and recapitulated those of human ST-EPN-RELA ependymomas [17, 21]. Thus, the oncogenic function of RELA^FUS1^ was reproducible in the lentiviral gene transfer system.

### CRISPR/Cas9-mediated gene rearrangement induces endogenous *C11orf95*-*RELA* fusion in human cultured cells

The *C11orf95*-*RELA* (*RELA*^*FUS*^) fusions are caused by a genomic rearrangement involving *C11orf95* and *RELA* loci on 11q in human ependymomas. This rearrangement involves a multitude of other genes in this region with unknown effects on tumorigenesis. Although forced expression of RELA^FUS1^ was sufficient to induce brain tumor formation in mice, the status of neighboring genes involved in the genomic events was not considered in previous models [17, 21]. The generation of endogenous oncogenic gene fusion events, subsequently resulting in the formation of lung or brain tumors, has previously been achieved via CRISPR/Cas9-mediated chromosomal rearrangements in mice in vivo [4, 11]. Therefore, we examined whether the oncogenic gene rearrangement, consequently resulting in endogenous *RELA*^FUS1^, can be experimentally reproduced.

**Fig. 2:**
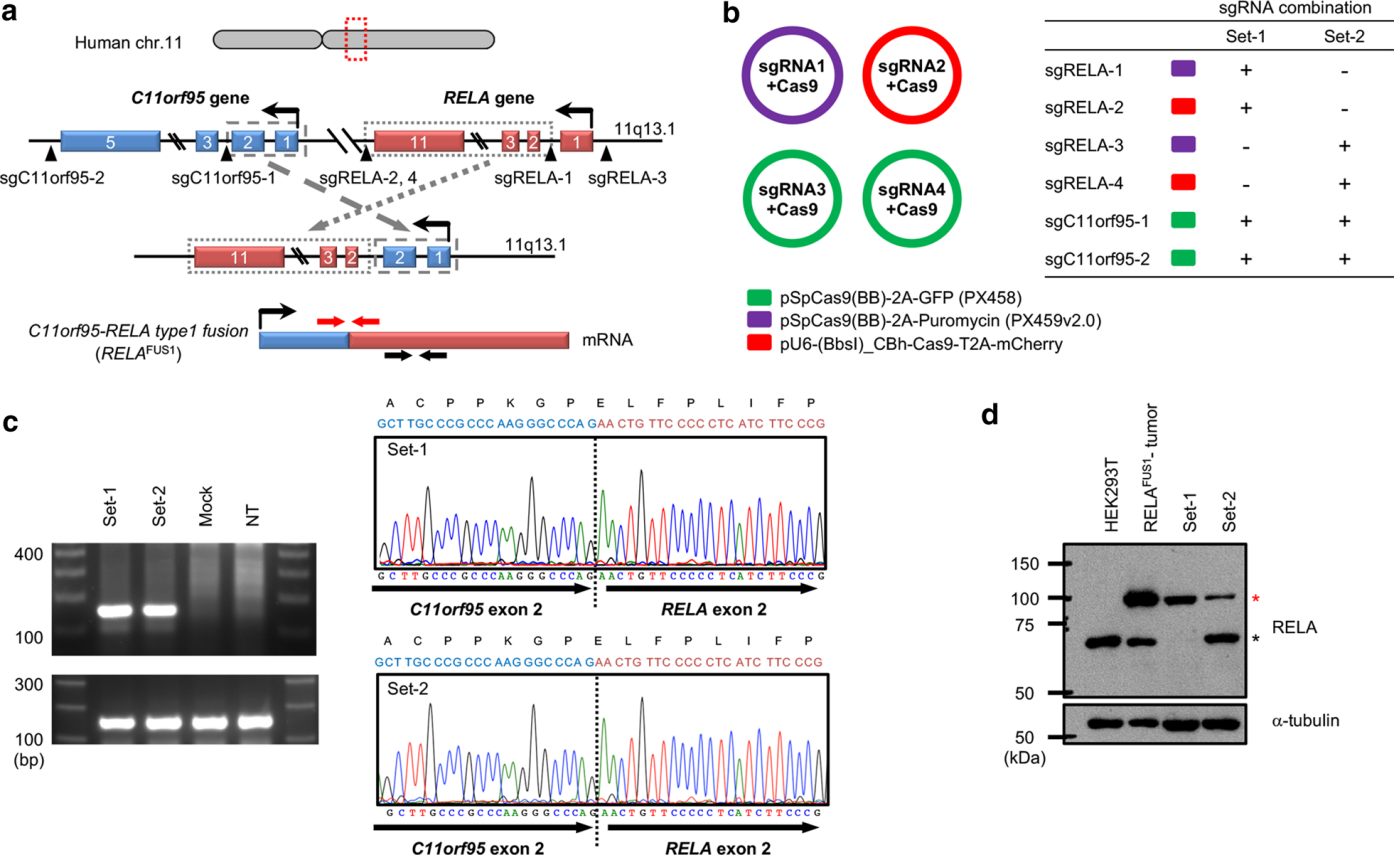
CRISPR/Cas9-mediated gene rearrangement induced endogenous *RELA*^FUS1^ in human cultured cells. **a** Experimental strategy for the CRISPR/Cas9-mediated gene rearrangement to induce endogenous *RELA*^FUS1^ in 293T cells. Blue and red boxes represent exons of the *C11orf95* and *RELA* genes, respectively. Black arrowheads indicate cleavage sites by the sgRNAs. Red arrows indicate PCR primer pairs for the fusion-junction detection. **b** sgRNA combination for the CRISPR/Cas9-mediated gene rearrangement in 293T cells. **c** RT-PCR detection of *RELA*^FUS1^ transcript in 293T cells. RNA was extracted from 293T cells that were transiently transfected with the sgRNA combination-1, 2 or mock vectors (left panel) The RNA was then subjected to RT-PCR analyses for C11orf95-RELA fusion junction (left-top) and intact RELA detection (left-bottom panel). The electropherograms of the Set-1 and 2 PCR products are shown in right-top and right-bottom panels, respectively. NT, non-treated parental 293T cells. **d** Western blot analysis for RELA^FUS1^ detection in the gene-edited 293T cells. The set-1 and set-2 sgRNAs were transiently transfected into 293T cells. Cell lysates were subjected to immunoblot blot analysis with the indicated antibodies. Upper (red) and lower (black asterisk) bands in the RELA antibody detection indicate RELA^FUS1^ and endogenous RELA protein, respectively. Cell lysate extracted from pTomo-RELA^FUS1^-induced brain tumor tissue serves as a positive control for RELA^FUS1^ protein expression

*RELA*^FUS1^ consists of the first two exons of *C11orf95* and exons two to eleven of *RELA* [21]. To reproduce the gene rearrangement giving rise to the *RELA*^*FUS1*^ transcript, we designed four sgRNAs (single guide RNAs) on the human *C11orf95* and *RELA* gene loci around the breakpoints observed in human ependymomas harboring *RELA*^FUS1^ (Fig. 2a) [21]. We then generated four expression vectors for these sgRNAs and transiently transfected them in two combinations into 293T cells (Fig. 2b). RT-PCR analysis across the fusion junction of *RELA*^FUS1^ detected the *RELA*^FUS1^ transcript in both sgRNA sets. The sequences were subsequently confirmed by Sanger sequencing of the PCR products (Fig. 2c). In addition, the presence of the RELA^FUS1^ protein in 293T cells carrying the rearranged gene loci could also be detected by Western Blot (Fig. 2d). Genomic PCR analyses revealed that the target sequences were cleaved through the CRISPR/Cas9 system as intended, thereby leading to the induction of the gene rearrangement (Additional file 7: Fig. S2A and B). These results suggested that the endogenous human *RELA*^FUS1^ gene can be experimentally generated by introducing the intended breakpoints in human cultured cells via CRISPR/Cas9 technology.

### CRISPR/Cas9-mediated gene rearrangement induces oncogenic *2700081O15Rik*-*Rela* fusion in mice

**Fig. 3:**
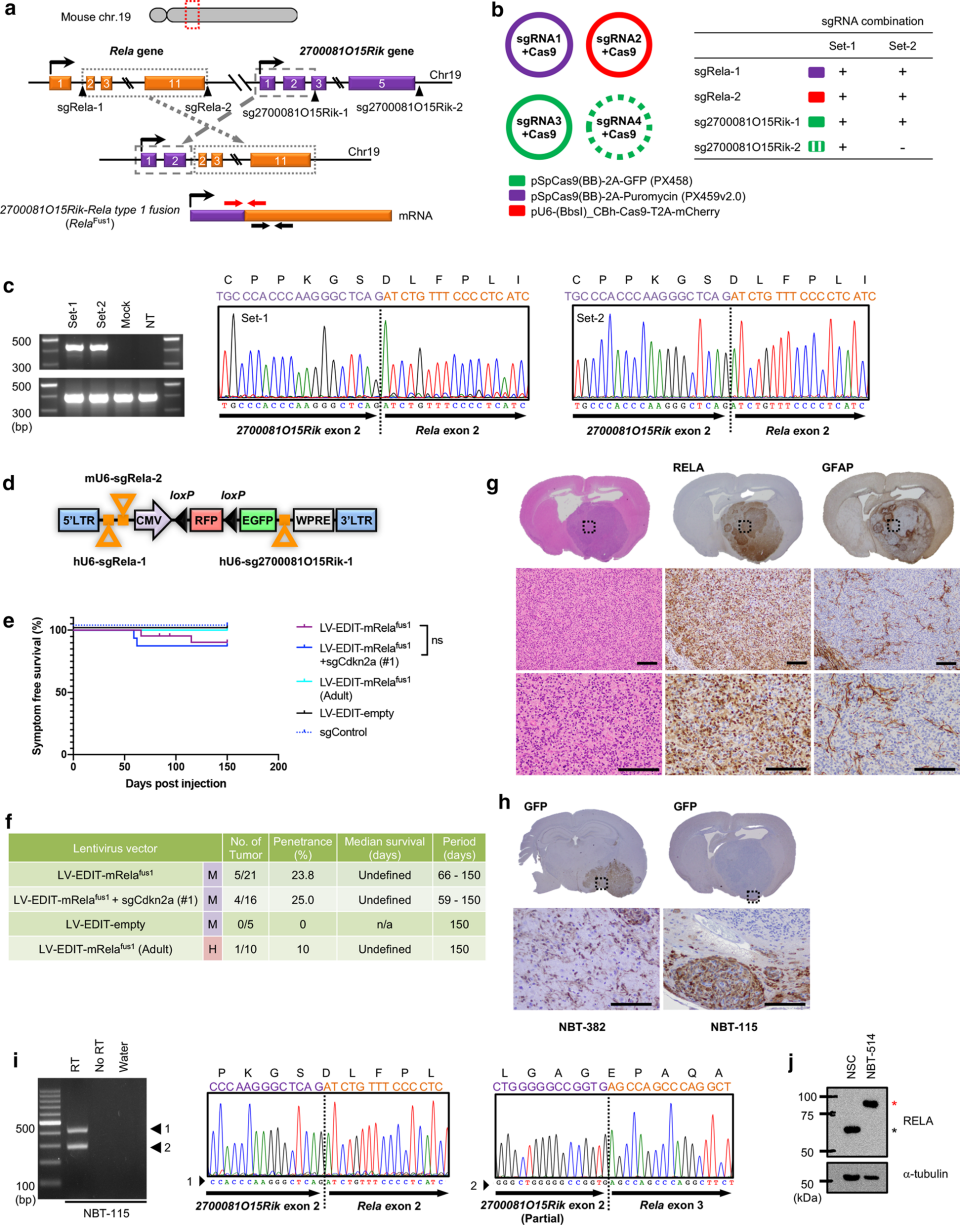
CRISPR/Cas9-mediated gene rearrangement generated induces oncogenic *2700081O15Rik*-*Rela* fusion in mice. **a** Experimental strategy for the CRISPR-Cas9-mediated gene rearrangement to induce m*Rela*^fus^. Orange and purple boxes represent exons of *Rela* and *2700081O15Rik* gene, respectively. Black arrowheads and Red arrows indicate cleavage sites by the sgRNAs and PCR primer pairs for the fusion-junction detection, respectively. **b** sgRNA combinations for the CRISPR/Cas9-mediated gene rearrangement in NIH3T3 cells. **c** RT-PCR detection of the m*Rela*^fus^ transcript in the NIH3T3 cells. RNA was extracted from NIH3T3 cells transiently transfected with the sgRNA combination-1, 2 or mock vectors (left panel) and then subjected to RT-PCR analyses for 2700081O15Rik-Rela fusion junction (left-top) and intact Rela detection (left-bottom panel). The electropherogram of the PCR products (Set-1 and 2) is shown in the middle and right panel. NT, non-treated parental NIH3T3 cells. **d** Schematic presentation of the pTomo-LV-EDIT-mRela^fus^ vector construct for simultaneous multiple sgRNA delivery into the mouse brain. mU6, murine U6 promoter. See also Additional file 6: Fig. S1A. **e** and **f** Kaplan–Meier survival curves and Summary of the LV-EDIT-mRela^fus^ vector injections. Lentiviruses were injected into neonatal pups brain in *Nestin*-*Cre*^+*/*−^*;Cag*-*Cas9*^+*/*+^ mice (n.s., not significant. M, medium titer; H, higher titer; n/a, not applicable). **g** and **h** Representative H&E and IHC stains for RELA, GFAP and GFP antibodies of the endogenous mouse *Rela* fusion-induced brain tumor in *Nestin*-*Cre*^+*/*−^*;Cag*-*Cas9*^+*/*+^ mice (n = 3). **i** RT-PCR detection of mRela^fus^ transcript in the LV-EDIT-mRela^fus^-induced brain tumor and electropherograms of the PCR products. Sanger sequencing analyses of the PCR products identified two types of in-frame fusion transcripts corresponding to human *RELA*^FUS1^ (middle panel) and *RELA*^FUS4^ (right panel) in this tumor (NBT-115). See also Additional file 8: Fig. S3C. **j** Western blot analysis in the mouse tumor-derived cell line (NBT-514) as shown in Additional file 8: Fig. S3C. Upper and lower bands in the RELA antibody detection indicate mRela^fus1^ and endogenous Rela protein, respectively

Successful detection of endogenous *RELA*^FUS1^ in human cultured cells encouraged us to examine whether this gene rearrangement can also be induced in mice, with subsequent formation of brain tumors. *Rela* is located upstream of the *2700081O15Rik* (the mouse homolog of human *C11orf95)* on chr19, a similar pattern to the human *RELA* and *C11orf95* genes on 11q (Figs. 2a and 3a). Therefore, to induce this gene rearrangement for the generation of the mouse *2700081O15Rik*-*Rela* fusion (m*Rela*^fus^), we designed sgRNAs in similar genomic positions compared to human *RELA*^FUS1^ (Figs. 2a and 3a). We then generated the vector constructs carrying sgRNAs with Cas9 and transiently transfected three (set-2) or four (set-1) sgRNAs into NIH3T3 cells (Fig. 3b). RT-PCR analysis detected m*Rela*^fus^ transcripts in both sgRNA sets and subsequent Sanger sequencing analyses of the PCR products confirmed the fusion junction sequence between exon 2 in *2700081O15Rik* and exon 2 in *Rela*, resulting in an in-frame fusion structurally corresponding to human *RELA*^FUS1^ (Fig. 3c). The full-length cDNA of m*Rela*^fus^ was isolated and validated by Sanger sequencing. Further, the endogenous mRela^fus^ protein was detected in cells transfected with the set-1 sgRNAs (Additional file 8: Fig. S3A).

Subsequently, we examined whether the endogenous m*Rela*^fus^ can induce brain tumors in mice. Given that the introduction of three breakpoints was sufficient to induce the fusion gene in vitro (Fig. 3c), we generated an all-in-one lentiviral vector including three sgRNAs (referred to as LV-EDIT-mRela^fus^) (Additional file 8: Fig. S3B) and co-injected the virus with or without the lentivirus for sg*Cdkn2a* (#1 target sequence) into the brain of neonatal pups and young adult mice in *Nestin*-*Cre*^+*/*−^*; Cag*-*Cas9*^+*/*+^ mice (Fig. 3d, e). Brain tumor formation was observed in one out of ten mice that were injected as young adults (Fig. 3e, f). By contrast, injection of neonatal pups led to brain tumor formation in 5 out of 21 mice around 60 days post-injection (Fig. 3e, f). We observed a considerably lower tumor incidence compared to our other model that relies on the lentiviral overexpression of *RELA*^*FUS1*^ (Figs. 1b and 3f). Additional *Cdkn2a* loss had no significant impact on tumor formation upon injection in neonatal pups in this model (Fig. 3e).

Mouse brain tumors induced with the endogenous m*Rela*^fus^ gene presented with similar morphological and histologic features compared to tumors induced by the gene transfer of the *RELA*^FUS1^ (Figs. 1e and 3g). Immunohistochemical staining with a RELA antibody detected intense nuclear immunoreactivity, indicating m*Rela*^fus^ protein expression. Although the m*Rela*^fus^ gene rearrangement was not necessarily specific to Nestin-expressing cells of origin in this model, immunostaining for GFP suggested that tumors might have arisen from Nestin positive neural stem cells in some cases (Fig. 3h). These immunohistochemical findings strongly suggests that tumors were induced by endogenous m*Rela*^fus^. We extracted RNA from m*Rela*^fus^ mouse brain tumors and were able to detect a fusion transcript corresponding to the human *RELA*^FUS1^ transcript by RT-PCR and subsequent sanger sequencing (Fig. 3i and Additional file 8: Fig. S3C). Another fusion transcript corresponding to human *RELA*^FUS4^ was also detected concomitantly in one mouse brain tumor sample (Fig. 3i) [21]. In addition, the mRela^fus1^ protein was detected by western blot in a cell line derived from one of the mouse brain tumors (Fig. 3j). These results support the notion that gene rearrangements are the primary mechanism to create the oncogenic *RELA*^*FUS1*^ fusion gene, serving as the direct driver of tumorigenesis in human RELA^FUS^ tumors.

### C11orf95-RELA fusion variants present diverse oncogenic potential

**Fig. 4:**
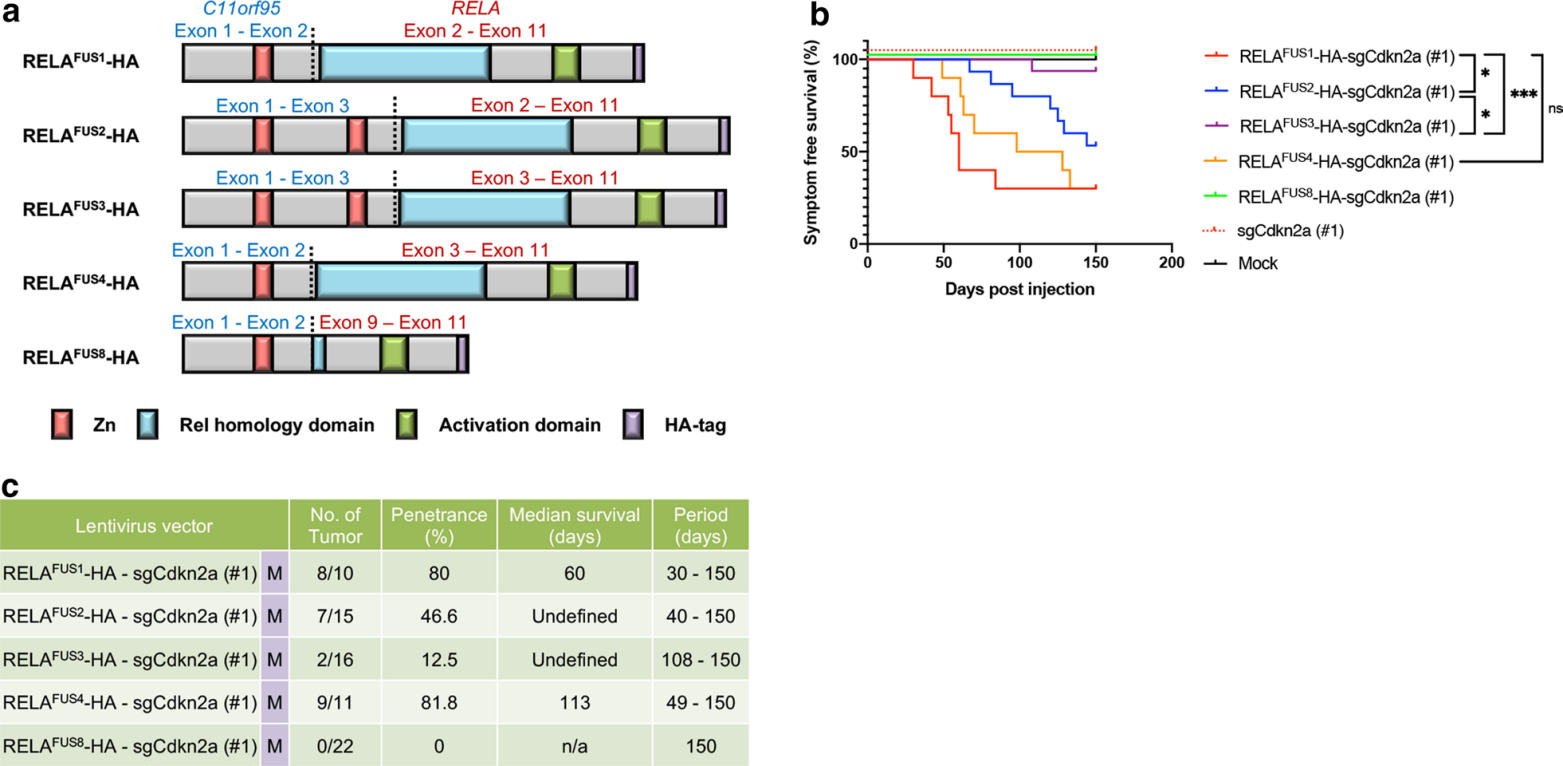
C11orf95-RELA fusion variants present diverse oncogenic potential. **a** Schematic presentation of the predicted protein products of various RELA^FUS^ variants with HA tag in the C-terminus in the pTomo-lentiviral vectors. Zn, zinc finger domain. **b** and **c** Kaplan–Meier survival curves and Summary of RELA^FUS^-HA-induced brain tumors. Lentiviruses were injected into neonatal pups brain in *Nestin*-*Cre*^+*/*−^*;Cag*-*Cas9*^+*/*+^ mice (n.s., not significant, *p < 0.05, ***p < 0.001. M, medium titer; n/a, not applicable)

Several *RELA*^FUS^ variants have been identified in human ST-EPN-RELA tumors [5, 8, 21]. The oncogenic potential of *RELA*^FUS1^ and *RELA*^FUS2^, the most frequent *RELA*^*FUS*^ variants, has previously been documented [17, 21]. However, the tumorigenic role of other less common *RELA*^FUS^ variants has not been fully characterized. Therefore, we investigated which of these *RELA*^FUS^ variants can induce brain tumor formation upon expression in neonatal pups using our lentiviral gene transfer system. *RELA*^FUS^ variants 5, 6, and 7 have a similar structure to *RELA*^FUS2^, apart from small intronic intervening sequences, and share the same functional domains. Therefore, we focused our study on *RELA*^FUS^ variants 2, 3 and 4 (Fig. 4a) [21]. In addition, we tested a small fusion transcript (referred to as *RELA*^FUS8^) lacking the Rel homology domain which we have recently identified (Fig. 4a) [8].

We generated pTomo-lentiviral vectors encoding for *RELA*^FUS^ variants 2, 3, and 4, along with a sgRNA targeting *Cdkn2a* (sgCdkn2a #1) (Additional file 9: Fig. S4A). Lentiviruses were injected into the brain of *Nestin*-*cre*^+*/*−^*;Cag*-*Cas9*^+*/*+^ neonatal pups. With the presence of *C11orf95* and *RELA* preserved functional domains in the different *RELA*^FUS^ variants, expression of *RELA*^*FUS2*-*4*^ induced brain tumor formation (Fig. 4b, c). Tumors generated by expression of *RELA*^FUS1^ and *RELA*^FUS4^ had the shortest tumor latencies. By contrast, tumors generated by expression of *RELA*^FUS2^ or *RELA*^FUS3^, which both possess two zinc finger domains of C11orf95, had significantly longer tumor latencies and lower tumor incidences, suggesting that an additional zinc finger domain might somehow negatively affect the tumorigenesis. There were no detectable lesions in the lentiviral injection of the *RELA*^FUS8^ and the control group (Fig. 4b, c). Thus, the Rel homology domain is likely essential for the oncogenic potential of the *RELA*^FUS^.

Histological features of these *RELA*^FUS^ variants were almost indistinguishable from those of the *RELA*^FUS1^-driven tumors and partly recapitulated characteristic histologic features of human ependymomas (Fig. 1e and Additional file 9: Fig. S4B-D). These results show that all *RELA*^FUS^ variants, except for the *RELA*^FUS8^ variant, are oncogenic with diverse tumor-forming potential.

### *YAP1* fusions induce brain tumors with different morphological and histologic features from *RELA*^FUS^-induced tumors in mice

**Fig. 5:**
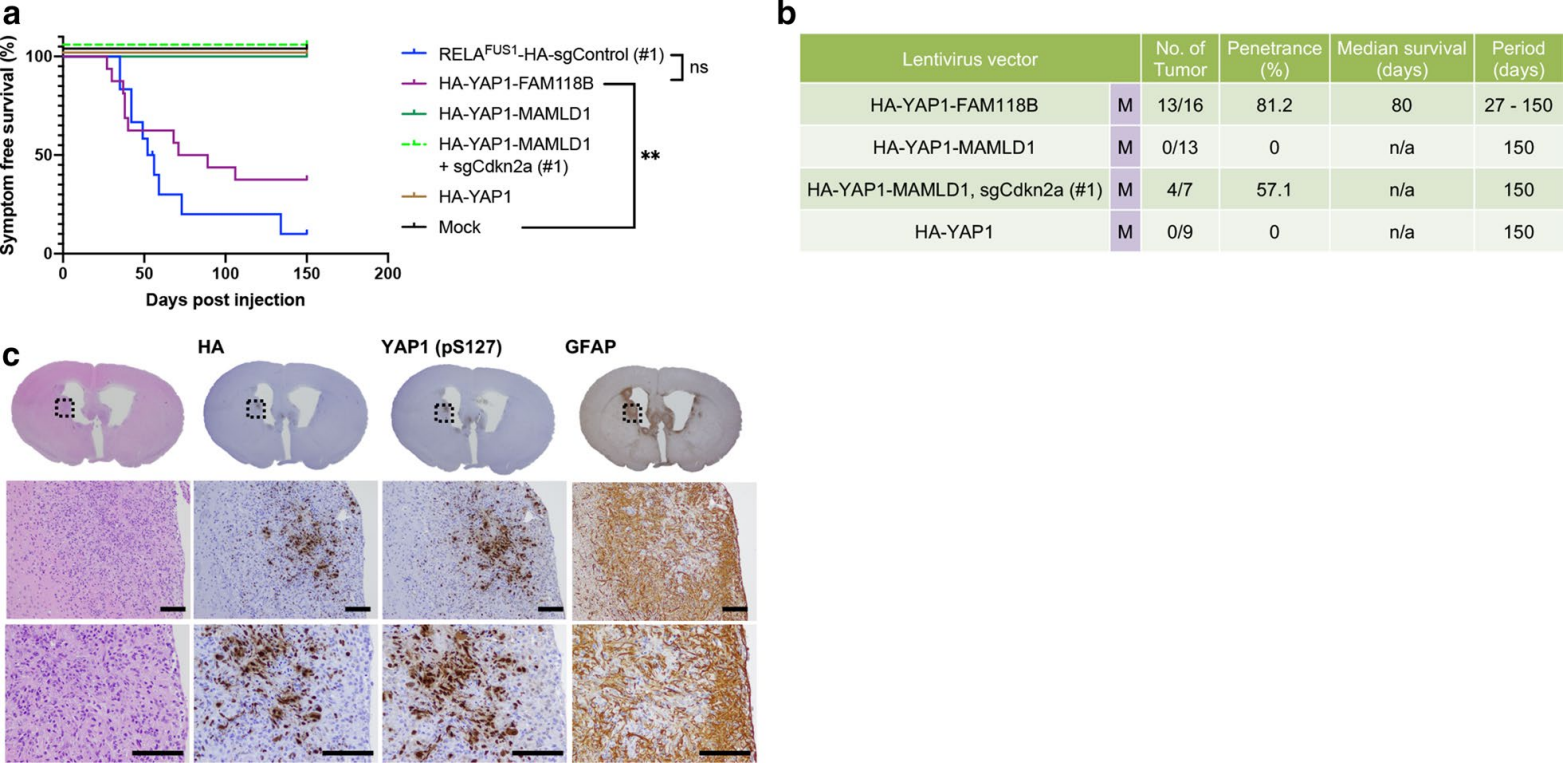
*YAP1* fusions induce brain tumors with different morphological and histologic features from *RELA*^FUS^-induced tumors in mice (**a** and **b**) Kaplan–Meier survival curves and Summary of YAP1 fusion-induced brain tumors. Lentiviruses were injected into neonatal pups brain in *Nestin*-*Cre*^+*/*−^*;Cag*-*Cas9*^+*/*+^ mice. For comparison, the survival curve of *RELA*^*FUS1*^-induced tumors as shown in Fig. 1a was depicted in the figure (n.s., not significant, **p < 0.01. M, medium titer; n/a, not applicable). **c** Representative H&E and IHC stains for HA tag, YAP1 and GFAP antibodies of the HA-YAP1-FAM118B-induced brain tumor in *Nestin*-*Cre*^+*/*−^*;Cag*-*Cas9*^+*/*+^ mice (n = 3)

ST-YAP1-EPN and ST-EPN-RELA tumors are recognized as clinically, genetically, and biologically distinct diseases [17, 19–21, 23]. To further understand the biology of ST-EPN-YAP1 tumors, we next examined the tumor-forming potential of recurrent *YAP1*-*MAMLD1* and *YAP1*-*FAM118B* fusions in our system (Additional file 10: Fig. S5A). We generated pTomo-*HA*-*YAP1*-*MAMLD1* and *HA*-*YAP1*-*FAM118B* lentiviral vectors and injected the lentiviruses into the brains of *Nestin*-*Cre*^+*/*−^*;Cag*-*Cas9*^+*/*+^ neonatal pups (Additional file 10: Fig. S5A and B). Injection of the *YAP1*-*FAM118B* fusion by itself caused the formation of mouse brain tumors with a similar latency to *RELA*^FUS1^ tumors, which is unlike the reported better clinical outcomes compared to *RELA*^FUS^ tumors in human patients (Fig. 5a, b) [20]. By contrast, no brain tumor formation was observed in the lentivirus injection with *YAP1*-*MAMLD1* (without additional Cdkn2a loss) (Fig. 5b), whereas small tumor or neoplastic lesions were detected upon additional co-injection of sgCdkn2a (Fig. 5b and Additional file 10: Fig. S5C). Intracranial lentiviral injection of *HA*-*YAP1* alone failed to induce brain tumor formation, reinforcing the necessity of the gene fusion partner for tumorigenesis (Fig. 5a, b) [19, 23].

*YAP1*-*FAM118B* fusion tumors mainly had a spindle-cell histomorphologic phenotype and tended to grow diffusely into the brain parenchyma, which is in contrast to the typical circumscribed growth pattern of *RELA*^FUS^-driven tumors (Fig. 5c). *YAP1*-*FAM118B* fusion tumors were rarely observed in the brain parenchyma and commonly localized to the adjacent intraventricular region, consistent with tumors arising from subventricular zone neural stem cells [24]. Pseudopalisading necrosis was sometimes detected, whereas we did not observe ependymal rosettes, ependymal canals, or perivascular pseudorosettes in these tumors. Immunohistochemical analyses for YAP1 and HA-tag revealed nuclear expression of the YAP1-FAM118B fusion protein in the tumor cells (Fig. 5c). YAP1 regulates gene expression as a transcriptional co-activator in the nucleus, thus nuclear localization likely occurs in the setting of persistent YAP1 pathway activation in these tumors [25]. Tumor cells were mostly negative for GFAP immunostaining. Excessive reactive astrocytes were commonly observed in the tumors as shown by extensive immunopositivity for GFAP adjacent to the HA-positive tumor cells (Fig. 5c). Overall, the *YAP1*-*FAM118B*-induced tumors appear to be high-grade spindle cell tumors rather than typical ependymoma. Similarly, small lesions induced with the YAP1-MAMLD1 fusion presented with an invasive phenotype that morphologically resembled the YAP1-FAM118B fusion tumors (Additional file 10: Fig. S5C). All together, these results provide evidence that the *YAP1*-*FAM118B* gene fusion is a potent oncogene capable of inducing brain tumors in mice.

## Discussion

In this study, we have successfully shown that the endogenous RELA fusion generated through targeted gene editing was sufficient to develop ependymoma-like brain tumors in mouse brains. Chromothripsis is one of the mechanisms to create a gene fusion event, but also simultaneously affects the status of neighboring genes on the involved chromosome [22]. Thus, *RELA*^FUS^ might have driven ependymoma formation in cooperation with numerous altered genes on 11q. However, it is not easy to determine which additional genes, if any, might be linked to *RELA*^*FUS*^-induced tumor formation. CRISPR/Cas9 system-mediated DNA double-strand breaks are repaired through Non-homologous end joining (NHEJ) more commonly and efficiently than the Homology Directed Repair (HDR) mechanism [13]. NHEJ is a primary repair mechanism for DNA double-strand break in chromothripsis [22]. Therefore, modeling gene rearrangements using the CRISPR/Cas9 technology seems to have reproduced at least one aspect of chromothripsis occurring in human ependymoma. Biological and histological similarities between m*Rela*^fus^ tumors and *RELA*^FUS^-induced tumors, as well as the successful detection of m*Rela*^fus^ transcript and protein, support the idea that m*Rela*^fus^ tumors are induced by endogenous m*Rela*^fus^, which is generated as a direct result of CRISPR/Cas9-mediated gene rearrangement. Thus, our model highly suggests that the biological consequence of genomic rearrangement in ependymomas is likely driven by the creation of novel splice junctions leading to the formation of oncogenic RELA fusion transcripts, while the potential influence of any neighboring gene(s) on the chromothriptic chromosome might be minimum, if at all, for tumorigenesis.

Mouse RELA fusion tumors recapitulated the phenotypic and histologic features of the human counterparts at various degrees as exemplified by the consistent branching capillary formation and intense nuclear immunopositivity of RELA. However, abundant perivascular pseudorosettes and GFAP-highlighted radial perivascular processes, the cardinal histologic features of ependymoma were not reproducibly observed. Furthermore, the spindle cell tumor morphology observed is unusual for ependymoma, except in the case of the tanycytic histologic variant of ependymoma. Our mouse model demonstrates that the ependymoma gene fusion products drive tumor formation in a specific context, but they do not necessarily recapitulate all aspects of human tumors. Thus, while our animal models provide important insights into the oncogenic mechanism of gene fusions, it is essential to recognize the limitations of these models and have the ability to refine these models based on accurate genetics and biology.

All tested *RELA*^FUS^ variants retaining the functional domain of RELA were oncogenic in our system. However, the tumor-forming potential of these variants were somewhat different, consistent with a previously published ex vivo model [21]. *RELA*^FUS1^ and *RELA*^FUS4^ formed tumors with faster latency and higher incidence than *RELA*^*FUS2*^ and *RELA*^FUS3^. Multiple fusion variants can be seen within a single ependymoma [8, 20, 21]. Although the complete biological significance of these fusion variants in ependymoma are not fully understood, the presence of multiple fusion variants might give rise to intra- or inter-tumor molecular heterogeneity, possibly engendering different patient outcomes and treatment sensitivity.

The *YAP1*-*MAMLD1* fusion was able to induce brain tumors in both prenatal and neonatal brains at varying efficacy [19, 23]. By contrast, the *YAP1*-*FAM118B* fusion was oncogenic by itself in neonatal, but not prenatal brains [19, 23]. ST-EPN-YAP1 tumors are commonly identified in children and approximately two-thirds are present in the infant population [20]. Although the age distribution of these fusion-positive cases is not determined yet due to their relatively rare occurrence, the cell of origin for *YAP1*-*FAM118B* fusion might be possibly different from that of *YAP1*-*MAMLD1* fusion tumors. Therefore, the oncogenic potential of *YAP1*-*FAM118B* fusion might rely on the temporal course of expression (e.g. during neonatal stage). Because inter-chromosomal rearrangement is indispensable for the creation of the *YAP1*-*MAMLD1* fusion, additional genetic alterations might be acquired and contribute to the flexible oncogenic potential of the *YAP1*-*MAMLD1* fusion. These findings indicate that the ST-EPN-YAP1 subgroup may be a heterogenous group of tumors which can be further subdivided according to their molecular features [8, 20]. Due to the unusual diffuse glioma or sarcoma like-histological findings in mouse *YAP1* fusion tumors, a larger cohort of human ST-EPN-YAP1 tumors will need to be investigated histologically and molecularly to determine the relevance of mouse *YAP1* fusion driven tumors as a model for human ependymoma.

Current molecular diagnosis of ST-EPN is focused on detection of the frequent splicing forms of *RELA*^*FUS*^. Given the oncogenic function of other *RELA*^FUS^ variants and *YAP1* fusions [19, 21, 23], detection of these less common fusion transcripts should be considered in routine surgical neuropathology practice. In addition to the diagnostic utility, detection of less common ependymoma gene fusions may have implications for potential therapeutic targeting strategies.

Genomic analyses of ependymomas have shed light onto some of the underlying tumor biology [8, 20, 21]. Nevertheless, the relative rarity of this tumor type still prevents the dynamic advance of ependymoma research and treatment because of the lack of currently available tumor derived-cell lines and patient-derived xenograft (PDX) models. Genetically accurate mouse models can provide a powerful platform for investigating such uncommon diseases. Genomic rearrangement models driven by an endogenous promoter that mirrors physiological gene expression have a great advantage as they are able to reproduce a more accurate biological situation compared to a gene transfer approach with an artificial promoter. Thus, we believe that our novel mouse ependymoma model has the potential to provide deeper insights into ependymoma biology, as well provide a platform to investigate therapeutic resistance and develop new treatment strategies for this aggressive disease [17, 19, 21].

## Conclusions

The presence of *RELA* and *YAP1* fusion transcripts is the hallmark of supratentorial ependymomas, and 11q chromosomal alterations are frequently associated with complex gene rearrangements. In order to gain a deeper understanding of ependymoma biology, it is essential to ascertain whether the presence of these fusion genes is sufficient for tumor formation or if additional abnormalities of neighboring genes involving in the genomic rearrangement are necessary. Our findings indicate that the biological function of chromosomal alterations in *RELA* fusion ependymomas is essentially to create a novel splice junction, consequently resulting in an oncogenic fusion gene. These findings are supported by the fact that simple overexpression of human variants of RELA fusions, without additional genetic alterations, induce tumor formation in mice. Thus, these observations not only support the need for precise neuropathological diagnosis of ependymoma molecular subgroups in the clinical setting, but also the exciting therapeutic potential for directly targeting specific fusion proteins found in supratentorial ependymoma.

## Supplementary information

**Additional file 1**. Supplementary materials and methods

**Additional file 2: Table S1**. List of vector constructs

**Additional file 3: Table S2**. List of sgRNA target sequence

**Additional file 4: Table S3**. List of primers

**Additional file 5: Table S4**. List of antibodies

**Additional file 6: Figure S1**. Lentiviral gene transfer of *C11orf95-RELA* type 1 fusion into mouse neural stem cells induces human ependymoma-like tumors (A) Schematic presentation of the pTomo-RELA^FUS1^-HA-sgRNA lentiviral vectors. Injection of the pTomo-lentivirus into the *Nestin-Cre*^*+/-*^*;Cag-Cas9*^*+/+*^ mouse brain induces RELA^FUS1^ expression specifically in the Nestin-expressing cells through the Cre-loxP system. The sgRNA targeting *Cdkn2a* or control sequence is expressed under the U6 promoter. LTR, long terminal repeat; CMV, human cytomegalovirus immediate early enhancer and promoter; RFP, red fluorescent protein; IRES, internal ribosome entry site; EGFP, enhanced green fluorescent protein; hU6, human U6 promoter; WPRE, woodchuck hepatitis virus posttranscriptional regulatory element (B) Western blot analysis for RELA^FUS1^-HA and p19ARF protein expression. NIH3T3 cells (left panel) or primary mouse embryonic fibroblasts (right panel) were infected with pTomo lentiviruses as indicated (left panel). Subsequently, pCAG-Cre vector was transiently transfected to induce Cre-meditated recombination in these cells. Cell lysates were then subjected to immunoblot analysis with the indicated antibodies. The upper (red) and lower (black asterisk) bands in the RELA antibody detection indicate RELA^FUS1^-HA and endogenous Rela protein, respectively. α-tubulin and β-actin were used as an internal control. (C) PCR genotyping of *Nestin-Cre*, *Cag-Cas9* and *Nestin-Cre*^*+/-*^*;CAG-Cas9*^*+/+*^ mice (D) qRT-PCR analysis of *Cdkn2a* expression in primary mouse embryonic fibroblasts infected with the pTomo-RELA^FUS1^-HA-sgCdkn2a virus or sgControl virus. Expression was normalized using *Rps18* as an internal control. Data represent Mean ± SEM with four samples per group. ***, P < 0.001. Data are representative of two independent experiments. (E) Sanger sequencing analyses of the colony-PCR products. Genomic DNAs were extracted from FFPE brain tumor tissues using a QIAamp DNA FFPE kit (Qiagen) according to the manufacturer’s protocol. The PCR products of the targeted loci were cloned into T-vector pMD20 (Takara). Each bacterial colony was PCR-amplified and sequenced (4 tumors, 5 clones each). The sgRNA sequence for *Cdkn2a* is shown in red and the 3’-PAM sequence is shown in green. Insertions or deletions are shown in blue. NBT-96, 100 and NBT-141, 164 denote DNA samples from the RELA^FUS1^-sgCdkn2a (#1) or sgControl (#1)-induced brain tumors, respectively. (F) Representative H&E and IHC analyses for HA tag, RELA and GFAP antibodies of the RELA^FUS1^-HA+sgCdkn2a (#1)-induced brain tumor in *Nestin-Cre*^*+/-*^*;CAG-Cas9*^*+/+*^ mice (n=3). Dashed boxes at the top panels denote the enlarged regions as shown at the bottom. Scale bars, 100 μm.

**Additional file 7: Figure S2**. CRISPR/Cas9-mediated gene rearrangement induces endogenous *C11orf95-RELA* fusion in human cultured cells. (A and B) Genomic PCR analyses in the gene-edited and parental 293T cells. DNAs were extracted from 293T cells transfected with set-1 (A) or 2 (B) sgRNAs and subjected to genomic PCR analysis. Blue and red boxes represent exons of the *C11orf95* and *RELA* gene, respectively. Black arrowheads and Red arrows indicate cleavage sites by the sgRNAs and the position of PCR primers designed to detect gene rearrangement, respectively. Primer pairs as AB, CD, EF, GH, IJ, and KL detect an unedited intact gene. Positive bands with primer pair A/F and A/J indicate successful gene rearrangement between RELA exon 2 and C11orf95 exon 2 in the set-1 combination and between RELA exon 1 and C11orf95 exon 2 in the set-2 combination, respectively. The RT-PCR and sequencing analysis in the gene-editing with set-2 sgRNAs revealed that RELA exon 1 served as an intronic non-coding sequence (Fig. S2B, second top panel, grey box).

**Additional file 8: Figure S3**. CRISPR/Cas9-mediated gene rearrangement generated induces oncogenic *2700081O15Rik-Rela* fusion in mice. (A) Western blot analysis in the NIH3T3 cells edited with the set-1 sgRNAs. The set-1 sgRNAs were transiently transfected into NIH3T3 cells, and GFP and RFP-positive cells were then selected using limiting dilution in puromycin containing media. Subsequently, cell lysates were subjected to immunoblot blot analysis with the indicated antibodies. Parental NIH3T3 cells were served as a control in this experiment. Upper (red) and lower (black arrow) bands in the RELA antibody detection indicate mRela^fus^ and endogenous Rela protein, respectively. α-tubulin was used as an internal control. (B) RT-PCR detection of mRela^fus^ transcript in the NIH3T3 cells. Cells were coinfected with the LV-EDIT-mRela^fus^ and lentiCRISPRv2 virus for Cas9 expression and then subjected to RT-PCR analysis (left panel). RT and no RT denotes RT-PCR analysis with or without reverse transcriptase enzyme, respectively. The electropherogram of the PCR product in subsequent Sanger sequencing analysis was shown in the right panel. (C) RT-PCR detection of mRela^fus^ transcript in the LV-EDIT-mRela^fus^-induced brain tumor (left panel) and electropherogram of the PCR product (right panel). Sanger sequencing analysis of the PCR product identified in-frame fusion transcript corresponding to human RELA^FUS1^ in this tumor (NBT-514).

**Additional file 9: Figure S4**. C11orf95-RELA fusion variants present diverse oncogenic potential (A) Western blot analysis for functional validation of pTomo-RELA^FUS^-HA-sgCdkn2a (#1) vector expression. NIH3T3 cells were infected with various pTomo lentiviruses as described. Subsequently, pCAG-Cre vector was transiently transfected to induce Cre-meditated recombination in the cells. Cell lysates were then subjected to immunoblot analysis with the indicated antibodies. Upper and lower bands in the RELA antibody detection indicate RELA^FUS^-HA protein and endogenous Rela protein, respectively. (B-D) Representative H&E and IHC analyses for HA tag, RELA and GFAP antibodies of the RELA^FUS2^-HA (B), RELA^FUS3^-HA (C) or RELA^FUS4^-HA (D) + sgCdkn2a (#1)-induced brain tumors in *Nestin-Cre*^*+/-*^*;CAG-Cas9*^*+/+*^ mice. Dashed boxes in the top panels denote the enlarged regions as shown in the bottom panels. Scale bars, 100 μm.

**Additional file 10: Figure S5**. *YAP1* fusions induces brain tumors with different morphological and histologic features from *RELA*^FUS^-induced tumors in mice. (A) Schematic presentation of the predicted protein products of YAP1 fusions and wild-type YAP1 with HA tag in the N-terminus in the pTomo-lentiviral vectors. TID, TEA domain-containing factor-interaction domain for TEAD binding; WW, protein–protein interaction domain; MAML, mastermind-like domain; Ser, serine-rich region; Pro, proline-rich region; TAD, transcriptional activation domain for TEAD. (B) Western blot analysis for pTomo-HA-YAP1 fusion vector expression. NIH3T3 cells were infected with various pTomo lentiviruses as described. Subsequently, pCAG-Cre vector was transiently transfected to induce Cre-meditated recombination in the cells. Cell lysates were then subjected to immunoblot analysis with the indicated antibodies. (C) Representative H&E images of the HA-YAP1-MAMLD1 + sgCdkn2a (#1)-induced brain tumors in *Nestin-Cre*^*+/-*^*;CAG-Cas9*^*+/+*^ mice. Dashed boxes in the top panels denote the enlarged regions as shown in the bottom panels. Scale bars, 100 μm.

## Abbreviations

RELA^FUS1^: C11orf95-RELA type 1 fusion
sgRNA: Single guide RNA
ST-EPN: Supratentorial ependymoma
mRela^fus^: 2700081O15Rik-Rela fusion

## Availability of data and materials

All the data and materials generated in this study are available from the corresponding authors on reasonable request.

## References

[1] Albers J, Danzer C, Rechsteiner M, Lehmann H, Brandt LP, Hejhal T, Catalano A, Busenhart P, Goncalves AF, Brandt S (2015). A versatile modular vector system for rapid combinatorial mammalian genetics. J Clin Investig.

[2] Cage TA, Clark AJ, Aranda D, Gupta N, Sun PP, Parsa AT, Auguste KI (2013). A systematic review of treatment outcomes in pediatric patients with intracranial ependymomas. J Neurosurg Pediatr.

[3] Chiou SH, Winters IP, Wang J, Naranjo S, Dudgeon C, Tamburini FB, Brady JJ, Yang D, Gruner BM, Chuang CH (2015). Pancreatic cancer modeling using retrograde viral vector delivery and in vivo CRISPR/Cas9-mediated somatic genome editing. Genes Dev.

[4] Cook PJ, Thomas R, Kannan R, de Leon ES, Drilon A, Rosenblum MK, Scaltriti M, Benezra R, Ventura A (2017). Somatic chromosomal engineering identifies BCAN-NTRK1 as a potent glioma driver and therapeutic target. Nat Commun.

[5] de Sousa GR, Marie SKN, Oba-Shinjo SM, Ramalho LNZ, Tone LG, Valera ET (2019). A novel type of C11orf95-LOC-RELA fusion in a grade II supratentorial ependymoma: report of a case with literature review. Child’s Nerv Syst ChNS Off J Int Soc Pediatr Neurosurg.

[6] Figarella-Branger D, Lechapt-Zalcman E, Tabouret E, Junger S, de Paula AM, Bouvier C, Colin C, Jouvet A, Forest F, Andreiuolo F (2016). Supratentorial clear cell ependymomas with branching capillaries demonstrate characteristic clinicopathological features and pathological activation of nuclear factor-kappaB signaling. Neuro-oncology.

[7] Friedmann-Morvinski D, Singer O (2013). Overexpression Models: lentiviral modeling of brain cancer. Curr Protoc Mouse Biol.

[8] Fukuoka K, Kanemura Y, Shofuda T, Fukushima S, Yamashita S, Narushima D, Kato M, Honda-Kitahara M, Ichikawa H, Kohno T (2018). Significance of molecular classification of ependymomas: C11orf95-RELA fusion-negative supratentorial ependymomas are a heterogeneous group of tumors. Acta Neuropathol Commun.

[9] Gururangan S, Fangusaro J, Young Poussaint T, Onar-Thomas A, Gilbertson RJ, Vajapeyam S, Gajjar A, Goldman S, Friedman HS, Packer RJ (2012). Lack of efficacy of bevacizumab + irinotecan in cases of pediatric recurrent ependymoma—a Pediatric Brain Tumor Consortium study. Neuro-oncology.

[10] Mack SC, Witt H, Piro RM, Gu L, Zuyderduyn S, Stutz AM, Wang X, Gallo M, Garzia L, Zayne K (2014). Epigenomic alterations define lethal CIMP-positive ependymomas of infancy. Nature.

[11] Maddalo D, Manchado E, Concepcion CP, Bonetti C, Vidigal JA, Han YC, Ogrodowski P, Crippa A, Rekhtman N, de Stanchina E (2014). In vivo engineering of oncogenic chromosomal rearrangements with the CRISPR/Cas9 system. Nature.

[12] Marumoto T, Tashiro A, Friedmann-Morvinski D, Scadeng M, Soda Y, Gage FH, Verma IM (2009). Development of a novel mouse glioma model using lentiviral vectors. Nat Med.

[13] Maruyama T, Dougan SK, Truttmann MC, Bilate AM, Ingram JR, Ploegh HL (2015). Increasing the efficiency of precise genome editing with CRISPR-Cas9 by inhibition of nonhomologous end joining. Nat Biotechnol.

[14] Merchant TE, Bendel AE, Sabin ND, Burger PC, Shaw DW, Chang E, Wu S, Zhou T, Eisenstat DD, Foreman NK (2019). Conformal radiation therapy for pediatric ependymoma, chemotherapy for incompletely resected ependymoma, and observation for completely resected, supratentorial ependymoma. J Clin Oncol Off J Am Soc Clin Oncol.

[15] Naito Y, Hino K, Bono H, Ui-Tei K (2015). CRISPRdirect: software for designing CRISPR/Cas guide RNA with reduced off-target sites. Bioinformatics.

[16] Ostrom QT, Cioffi G, Gittleman H, Patil N, Waite K, Kruchko C, Barnholtz-Sloan JS (2019). CBTRUS statistical report: primary brain and other central nervous system tumors diagnosed in the United States in 2012–2016. Neuro-oncology.

[17] Ozawa T, Arora S, Szulzewsky F, Juric-Sekhar G, Miyajima Y, Bolouri H, Yasui Y, Barber J, Kupp R, Dalton J (2018). A de novo mouse model of C11orf95-RELA fusion-driven ependymoma identifies driver functions in addition to NF-kappaB. Cell Rep.

[18] Pajtler KW, Mack SC, Ramaswamy V, Smith CA, Witt H, Smith A, Hansford JR, von Hoff K, Wright KD, Hwang E (2017). The current consensus on the clinical management of intracranial ependymoma and its distinct molecular variants. Acta Neuropathol.

[19] Pajtler KW, Wei Y, Okonechnikov K, Silva PBG, Vouri M, Zhang L, Brabetz S, Sieber L, Gulley M, Mauermann M (2019). YAP1 subgroup supratentorial ependymoma requires TEAD and nuclear factor I-mediated transcriptional programmes for tumorigenesis. Nat Commun.

[20] Pajtler KW, Witt H, Sill M, Jones DT, Hovestadt V, Kratochwil F, Wani K, Tatevossian R, Punchihewa C, Johann P (2015). Molecular classification of ependymal tumors across all CNS compartments, histopathological grades, and age groups. Cancer Cell.

[21] Parker M, Mohankumar KM, Punchihewa C, Weinlich R, Dalton JD, Li Y, Lee R, Tatevossian RG, Phoenix TN, Thiruvenkatam R (2014). C11orf95-RELA fusions drive oncogenic NF-kappaB signalling in ependymoma. Nature.

[22] Stephens PJ, Greenman CD, Fu B, Yang F, Bignell GR, Mudie LJ, Pleasance ED, Lau KW, Beare D, Stebbings LA (2011). Massive genomic rearrangement acquired in a single catastrophic event during cancer development. Cell.

[23] Szulzewsky F, Arora S, Hoellerbauer P, King C, Nathan E, Chan M, Cimino PJ, Ozawa T, Kawauchi D, Pajtler KW (2020). Comparison of tumor-associated YAP1 fusions identifies a recurrent set of functions critical for oncogenesis. Genes Dev.

[24] Taylor MD, Poppleton H, Fuller C, Su X, Liu Y, Jensen P, Magdaleno S, Dalton J, Calabrese C, Board J (2005). Radial glia cells are candidate stem cells of ependymoma. Cancer Cell.

[25] Tremblay AM, Missiaglia E, Galli GG, Hettmer S, Urcia R, Carrara M, Judson RN, Thway K, Nadal G, Selfe JL (2014). The Hippo transducer YAP1 transforms activated satellite cells and is a potent effector of embryonal rhabdomyosarcoma formation. Cancer Cell.

[26] Vidigal JA, Ventura A (2015). Rapid and efficient one-step generation of paired gRNA CRISPR-Cas9 libraries. Nat Commun.

[27] Zuckermann M, Hovestadt V, Knobbe-Thomsen CB, Zapatka M, Northcott PA, Schramm K, Belic J, Jones DT, Tschida B, Moriarity B (2015). Somatic CRISPR/Cas9-mediated tumour suppressor disruption enables versatile brain tumour modelling. Nat Commun.

