# Supplementary material for: Phenotypic characterization with somatic genome editing and gene transfer reveals the diverse oncogenicity of ependymoma fusion genes
Source: Acta Neuropathol Commun. 2020 Nov 23;8:203. doi: 10.1186/s40478-020-01080-8 (PMC7684901; doi:10.1186/s40478-020-01080-8)
Supplement: Supplementary file 1 — Additional file 1. Supplementary materials and methods [file 40478_2020_1080_MOESM1_ESM.docx]

**SUPPLEMENTARY METHODS**

**Phenotypic characterization with somatic genome editing and gene transfer reveal the diverse oncogenicity of ependymoma fusion genes**

Mutsumi Takadera, Kaishi Satomi, Frank Szulzewsky, Patrick J Cimino, Eric C. Holland, Tetsuya Yamamoto, Koichi Ichimura, Tatsuya Ozawa

**Plasmids**

pTomo lentiviral vector was a kind gift from Dr. Inder Verma (Addgene #26291). RCAS-RELA^FUS1^-HA, RCAS-HA-YAP1, RCAS-HA-YAP1-MAMLD1, and RCAS-HA-YAP1-FAM118B vectors were kindly provided by Dr. Eric C. Holland at the Fred Hutchinson Cancer Research Center. For generation the pTomo-RELA^FUS1-4 and 8^ vectors, each coding sequence (CDS) was PCR-amplified from RCAS-RELA^FUS1^-HA vector or human ependymoma tumor tissues and subcloned into pTA2 (TAKARA) or pCR4Blunt-TOPO vector (life technologies). Subsequently, CDSs for the human RELA^FUS^ (C-terminus HA-tagged) were further PCR-amplified and inserted into the *Bam*HI site of the pTomo vector. For the introduction of gene rearrangement and *Cdkn2a* gene knockout, the hU6-sgRNA fragments with the oligonucleotides of the relevant target sequence were transferred to the pTomo-RELA^FUS^ vectors using the *Bsr*GI and *Sal*I sites or the *Hpa*I site as shown Fig. 1A and 3D. The sgRNA sequence targeting *Cdkn2a* and control were previously described [1, 5]. Other sgRNAs were designed using the CRISPRDirect tool [3].

For generation of pTomo-HA-YAP1 vectors, *Bam*HI-HA-YAP1-*Pme*I-*Bgl*II sequence PCR-amplified from the RCAS-HA-YAP1 vector was inserted into the *Bam*HI site of the pTomo vector; pTomo-HA-YAP1-FAM11B vector was generated by replacing the HA-YAP1 fragment to the *Bam*HI-HA-YAP1-FAM11B-*Swa*I fragment from RCAS-HA-YAP1-FAM11B vector using the *Bam*HI and *Pme*I sites in the pTomo-HA-YAP1 vector; pTomo-HA-YAP1-MAMLD1 vector was generated by inserting the *Hinc*II-HA-YAP1-MAMLD1-*Pac*I fragment from RCAS-HA-YAP1-MAMLD1 using the *Eco*RV and *Pac*I sites into the modified-pTomo vector added *Pac*I site in the cloning site.

pSpCas9(BB)-2A-GFP (PX458), pSpCas9(BB)-2A-Puro (PX459) V2.0 (Addgene #48138 and #62988, a kind gift from Feng Zhang), and pU6-(*Bbs*I)_CBh-Cas9-T2A-mCherry (Addgene #64324, a kind gift from Ralf Kuehn) were used for *in vitro* sgRNA validation. These plasmids were digested with *Bbs*I and ligated with sgRNA oligonucleotides targeting *C11orf95*, *RELA*, *YAP1*, *FAM118B*, *MAMLD1*, *2700081O15Rik* and *Rela* genes according to the original online protocol of the Zhang lab (<https://media.addgene.org/cms/filer_public/e6/5a/e65a9ef8-c8ac-4f88-98da-3b7d7960394c/zhang-lab-general-cloning-protocol.pdf>).

For generation of LV-EDIT-mRela^fus^ vector, N-terminus 3xFlag-tagged EGFP cDNA was initially PCR-amplified from the PX458 and replaced it with the IRES-EGFP sequence in the pTomo vector. To prevent an unintended lentiviral recombination, we used the human U6 (hU6) and murine U6 (mU6) promoters for the U6-sgRNA constructs located nearby. Briefly, for cloning of the tandem U6-gRNA-Cas9 constructs, mU6-gRNA construct was generated from pDonor-mU6 (Addgene #69350, a kind gift from Andrea Ventura) by using the Gibson Assembly Master Mix (New England Biolabs, Cat. E2611L) [4]. The Gibson reaction was then digested with *Bbs*I at 37°C for 3 hours. The linearized fragment containing the paired gRNA, the mU6 promoter, and the gRNA scaffold were gel-purified and cloned into the PX459 V2.0 and then into the pTomo-GFP vector. All plasmids were confirmed by Sanger sequencing analyses (ABI3730xl). All vectors and sgRNA target sequences used in this study are listed in Supplementary Table S1 and S2.

**Lentivirus production, concentration, titration**

All lentiviral viruses were produced in 293T cells by transient transfection of the transfer vector, the packaging construct psPAX2 (Addgene #12260) and the vesicular stomatitis virus envelope-expressing construct pMD2.G (Addgene #12259) using X-tremeGENE9 (Roche). After 24 hours of transfection, the culture media were replaced with fresh culture media and then continued viral production. The viral supernatant was harvested at 48, 72, and 96 hours post-transfection and concentrated by the Lenti-X concentrator (Clontech). Viral titers were determined by the Lenti-X qRT-PCR Titration Kit according to the manufacture’s protocol (Clontech). The viruses were aliquoted and stored at -80°C freezer.

**Mouse and Genotyping**

We maintained the colonies of *Nestin-Cre* strain as heterozygotes and *Cag-Cas9* strain as homozygotes in the *C57BL/6J* background, respectively. *Nestin-cre^+/-^*; *Cag-Cas9^+/+^* mice were obtained by crossing *Nestin-cre* mice to *Cag-Cas9* mice. PCR genotyping was carried out on DNA extracted from mouse tails with the alkaline lysis method as follows. Mouse tails were lysed in 180 μl of 50 mM NaOH for 10 min at 95 °C. Then, lysates were neutralized with 20 μl of 1 M Tris-HCl, pH 8.0 and spun at 12,000 rpm for 10 min. PCR reaction was performed in a 12.5-μl reaction volume under the following condition: 6.3 μl of KOD ONE master mix (TOYOBO), three or four primer pairs (0.3 μM each), 2 μl of the lysate and autoclaved distilled water up to 12.5 μl. The protocol was denaturation for 10 sec at 98°C, annealing for 5 sec at 60 °C, and extension for 1 sec at 68 °C for 30 cycles. Subsequently, the PCR products were resolved by electrophoresis on 2 % agarose gel with GelRed Nucleic Acid Gel Stain (Biotium) (also see Fig. S1A). Primer sequences are listed in Table S3 [2].

**Generation of Mouse Brain Tumors**

For the lentivirus delivery into the young adult mouse brain, 3-5 weeks old mice were anesthetized with a ketamine-xylazine solution. Then, two microliters of lentiviruses (Lower titer group: 2 μl of 4.65 $\times$ 10^10^ copies/ml or High titer group: 2 μl of 9.3 × 10^10^ copies/ml) were stereotactically injected with a 33-gauge Hamilton syringe at the 0.4 μl/min speed. The following coordinates to targeting subventricular zone (SVZ) (mm posterior, lateral and dorsal to bregma) were used for body weight ≥ 10g (1.5, 2.0, 2.3) and body weight < 10g (1.2, 1.6, 1.85), respectively. After the injection, mice were placed on a 37 °C heating plate until they fully recovered and were returned to their cages. For the lentivirus delivery into the neonatal brain, one or two microliter(s) of lentiviruses (Lower titer group: 1 μl of 4.65 $\times$ 10^10^ copies/ml or Medium titer group: 1 μl of 9.3 $\times$ 10^10^ copies/ml) was injected into newborn pups brains within 3 days after birth. To simultaneously inactivate the *Cdkn2a* gene, 0.5 μl of the lentivirus for sgCdkn2a (#1) (9.3 × 10^10^ copies/ml) was co-injected with one microliter of the relevant lentivirus. The mice were monitored until they developed symptoms of disease, such as lethargy, macrocephaly, jumping, poor grooming, weight loss, dehydration, seizures and paralysis, or until five months after the lentivirus injection. Kaplan-Meier analysis demonstrating symptom-free survival of mouse brain tumors was performed using log-rank test in the GraphPad Prism 8 software. A value of p < 0.05 was considered significant in this study.

**qRT-PCR**

Total RNAs were extracted from cultured cells and brain tumor tissues using a miRNeasy Mini kit (Qiagen) according to the manufacturer’s protocol. cDNAs were synthesized with the Superscript IV VILO Master Mix (Invitrogen) according to the manufacturer’s protocol and used for subsequent PCR amplification. Quantitative-PCR (qRT-PCR) reaction was performed with the KOD SYBR qPCR Mix (Toyobo) on the CFX96 Real-Time PCR Detection System (Bio-Rad). The ΔΔCt method was used to calculate the relative *Cdkn2a* gene expression normalized to the reference gene (*Rps18*) with the CFX Manager Software (Bio-Rad). Data of qRT-PCR analysis are presented in bar graphs as mean and SE, except otherwise indicated. Results were analyzed by unpaired two-tailed Student’s t tests in the GraphPad Prism 8 software. Primer sequences are listed in Table S3.

**H&E Staining and Immunohistochemistry**

Brain tumor tissue samples were fixed in 10% formalin, paraffin-embedded and cut in 3 μm sections. For histopathological analysis, sections were stained with hematoxylin and eosin (H&E) carried out according to the routine institutional protocols. For immunohistochemical staining of mouse brains, tissue sections were deparaffinized in Hemo-De (Falma) and re-hydrated through a series of graded ethanol until water. Antigen retrieval was performed using 10 mM Citrate buffer pH 6.0 (Muto). Endogenous peroxidase was blocked with 3 % hydrogen peroxide and the slides were then incubated in blocking solution (normal 5 % goat serum in TBST). Primary antibodies were incubated with SignalStain Antibody Diluent (Cell Signaling Technology #8112). SignalStain Boost Detection Reagent (Cell Signaling Technology #8114) and SignalStain DAB Substrate Kit (Cell Signaling Technology #8059) was used as the detection system and the substrate according to the manufacturer’s protocol, respectively. Slides were finally counterstained with hematoxylin, dehydrated and mounted. Antibodies used in this study were listed in Table S4.

**SUPPLEMENTARY REFERENCES**

1 Albers J, Danzer C, Rechsteiner M, Lehmann H, Brandt LP, Hejhal T, Catalano A, Busenhart P, Goncalves AF, Brandt Set al (2015) A versatile modular vector system for rapid combinatorial mammalian genetics. The Journal of clinical investigation 125: 1603-1619 Doi 10.1172/JCI79743

2 Chiou SH, Winters IP, Wang J, Naranjo S, Dudgeon C, Tamburini FB, Brady JJ, Yang D, Gruner BM, Chuang CHet al (2015) Pancreatic cancer modeling using retrograde viral vector delivery and in vivo CRISPR/Cas9-mediated somatic genome editing. Genes Dev 29: 1576-1585 Doi 10.1101/gad.264861.115

3 Naito Y, Hino K, Bono H, Ui-Tei K (2015) CRISPRdirect: software for designing CRISPR/Cas guide RNA with reduced off-target sites. Bioinformatics 31: 1120-1123 Doi 10.1093/bioinformatics/btu743

4 Vidigal JA, Ventura A (2015) Rapid and efficient one-step generation of paired gRNA CRISPR-Cas9 libraries. Nat Commun 6: 8083 Doi 10.1038/ncomms9083

5 Zuckermann M, Hovestadt V, Knobbe-Thomsen CB, Zapatka M, Northcott PA, Schramm K, Belic J, Jones DT, Tschida B, Moriarity Bet al (2015) Somatic CRISPR/Cas9-mediated tumour suppressor disruption enables versatile brain tumour modelling. Nat Commun 6: 7391 Doi 10.1038/ncomms8391
