# Supplementary figures and images for: Phenotypic characterization with somatic genome editing and gene transfer reveals the diverse oncogenicity of ependymoma fusion genes
Source: Acta Neuropathol Commun. 2020 Nov 23;8:203. doi: 10.1186/s40478-020-01080-8 (PMC7684901; doi:10.1186/s40478-020-01080-8)

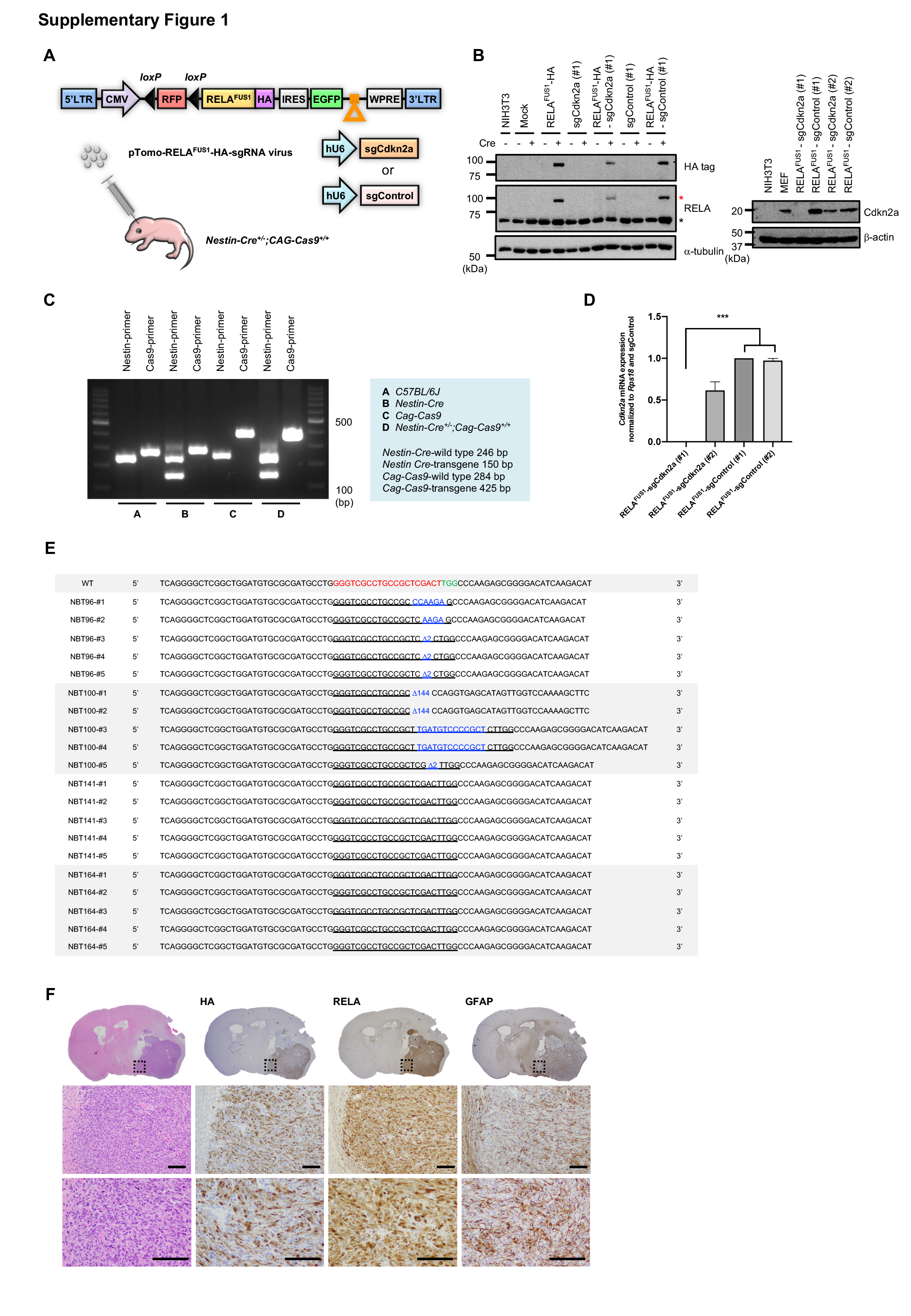

Supplement: Supplementary file 6 — Additional file 6: Figure S1. Lentiviral gene transfer of C11orf95-RELA type 1 fusion into mouse neural stem cells induces human ependymoma-like tumors (A) Schematic presentation of the pTomo-RELAFUS1-HA-sgRNA lentiviral vectors. Injection of the pTomo-lentivirus into the Nestin-Cre+/-;Cag-Cas9+/+ mouse brain induces RELAFUS1 expression specifically in the Nestin-expressing cells through the Cre-loxP system. The sgRNA targeting Cdkn2a or control sequence is expressed under the U6 promoter. LTR, long terminal repeat; CMV, human cytomegalovirus immediate early enhancer and promoter; RFP, red fluorescent protein; IRES, internal ribosome entry site; EGFP, enhanced green fluorescent protein; hU6, human U6 promoter; WPRE, woodchuck hepatitis virus posttranscriptional regulatory element (B) Western blot analysis for RELAFUS1-HA and p19ARF protein expression. NIH3T3 cells (left panel) or primary mouse embryonic fibroblasts (right panel) were infected with pTomo lentiviruses as indicated (left panel). Subsequently, pCAG-Cre vector was transiently transfected to induce Cre-meditated recombination in these cells. Cell lysates were then subjected to immunoblot analysis with the indicated antibodies. The upper (red) and lower (black asterisk) bands in the RELA antibody detection indicate RELAFUS1-HA and endogenous Rela protein, respectively. α-tubulin and β-actin were used as an internal control. (C) PCR genotyping of Nestin-Cre, Cag-Cas9 and Nestin-Cre+/-;CAG-Cas9+/+ mice (D) qRT-PCR analysis of Cdkn2a expression in primary mouse embryonic fibroblasts infected with the pTomo-RELAFUS1-HA-sgCdkn2a virus or sgControl virus. Expression was normalized using Rps18 as an internal control. Data represent Mean ± SEM with four samples per group. ***, P < 0.001. Data are representative of two independent experiments. (E) Sanger sequencing analyses of the colony-PCR products. Genomic DNAs were extracted from FFPE brain tumor tissues using a QIAamp DNA FFPE kit (Qiagen) according to the man [file 40478_2020_1080_MOESM6_ESM.tif]

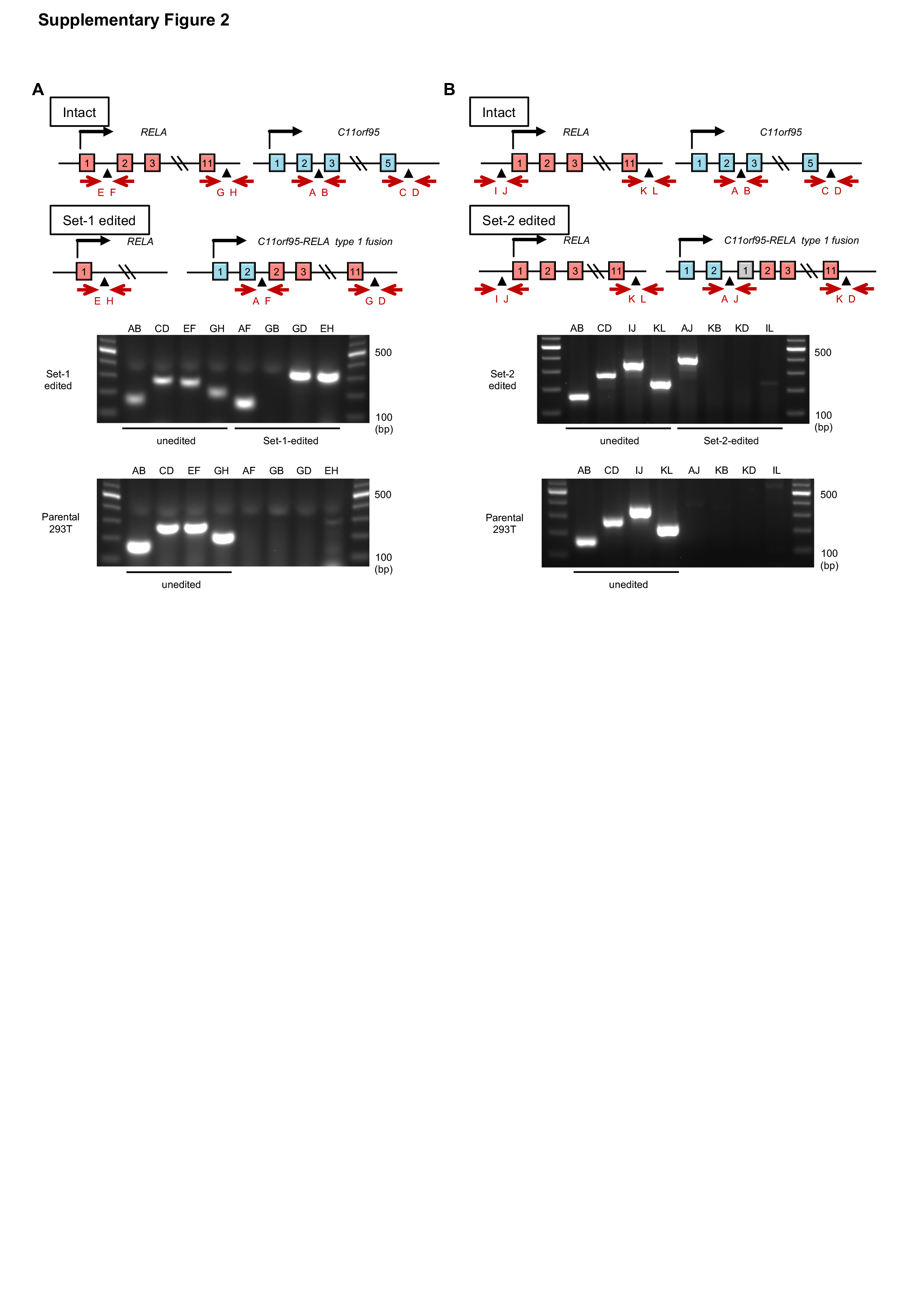

Supplement: Supplementary file 7 — Additional file 7: Figure S2. CRISPR/Cas9-mediated gene rearrangement induces endogenous C11orf95-RELA fusion in human cultured cells. (A and B) Genomic PCR analyses in the gene-edited and parental 293T cells. DNAs were extracted from 293T cells transfected with set-1 (A) or 2 (B) sgRNAs and subjected to genomic PCR analysis. Blue and red boxes represent exons of the C11orf95 and RELA gene, respectively. Black arrowheads and Red arrows indicate cleavage sites by the sgRNAs and the position of PCR primers designed to detect gene rearrangement, respectively. Primer pairs as AB, CD, EF, GH, IJ, and KL detect an unedited intact gene. Positive bands with primer pair A/F and A/J indicate successful gene rearrangement between RELA exon 2 and C11orf95 exon 2 in the set-1 combination and between RELA exon 1 and C11orf95 exon 2 in the set-2 combination, respectively. The RT-PCR and sequencing analysis in the gene-editing with set-2 sgRNAs revealed that RELA exon 1 served as an intronic non-coding sequence (Fig. S2B, second top panel, grey box). [file 40478_2020_1080_MOESM7_ESM.tif]

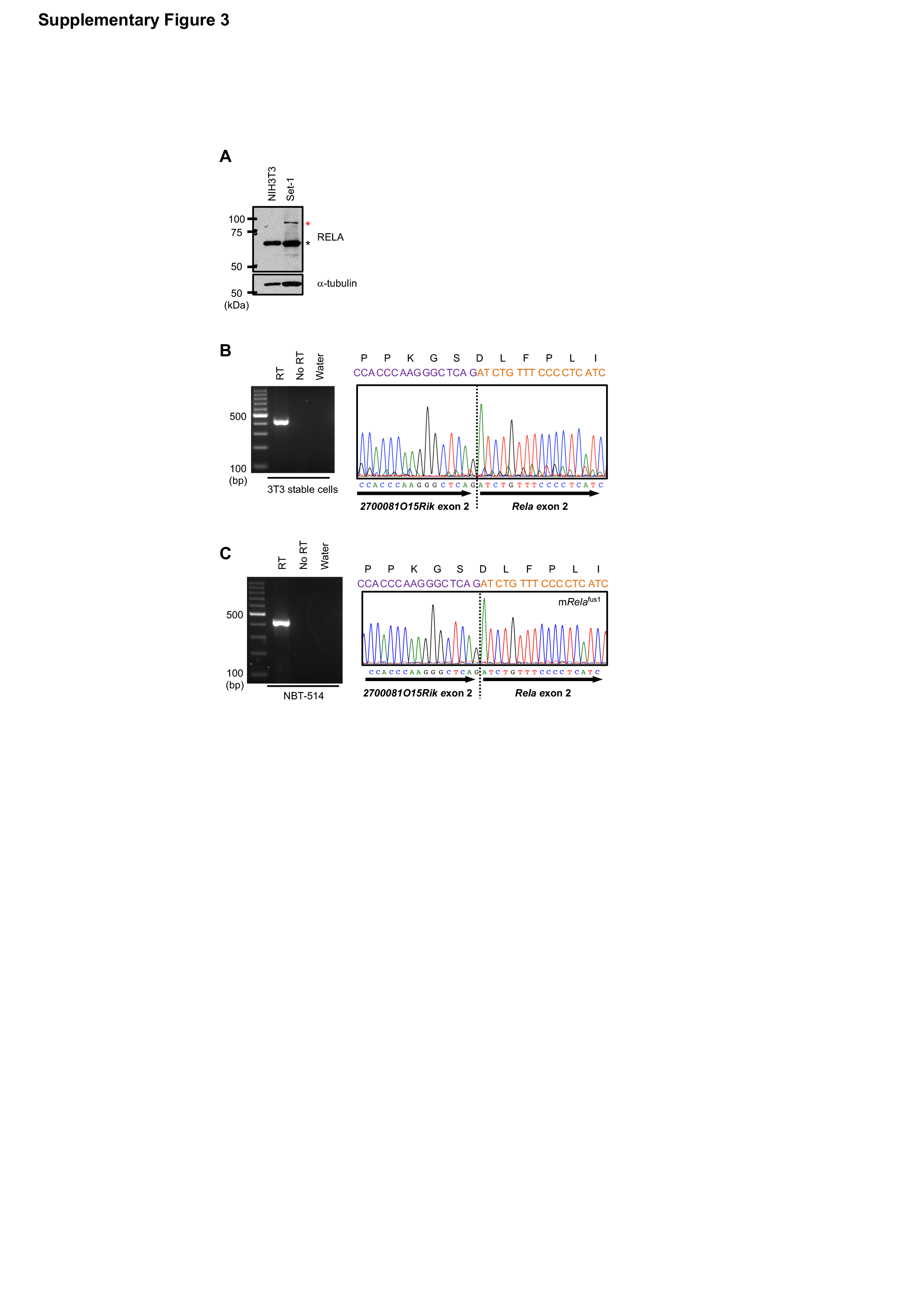

Supplement: Supplementary file 8 — Additional file 8: Figure S3. CRISPR/Cas9-mediated gene rearrangement generated induces oncogenic 2700081O15Rik-Rela fusion in mice. (A) Western blot analysis in the NIH3T3 cells edited with the set-1 sgRNAs. The set-1 sgRNAs were transiently transfected into NIH3T3 cells, and GFP and RFP-positive cells were then selected using limiting dilution in puromycin containing media. Subsequently, cell lysates were subjected to immunoblot blot analysis with the indicated antibodies. Parental NIH3T3 cells were served as a control in this experiment. Upper (red) and lower (black arrow) bands in the RELA antibody detection indicate mRelafus and endogenous Rela protein, respectively. α-tubulin was used as an internal control. (B) RT-PCR detection of mRelafus transcript in the NIH3T3 cells. Cells were coinfected with the LV-EDIT-mRelafus and lentiCRISPRv2 virus for Cas9 expression and then subjected to RT-PCR analysis (left panel). RT and no RT denotes RT-PCR analysis with or without reverse transcriptase enzyme, respectively. The electropherogram of the PCR product in subsequent Sanger sequencing analysis was shown in the right panel. (C) RT-PCR detection of mRelafus transcript in the LV-EDIT-mRelafus-induced brain tumor (left panel) and electropherogram of the PCR product (right panel). Sanger sequencing analysis of the PCR product identified in-frame fusion transcript corresponding to human RELAFUS1 in this tumor (NBT-514). [file 40478_2020_1080_MOESM8_ESM.tif]

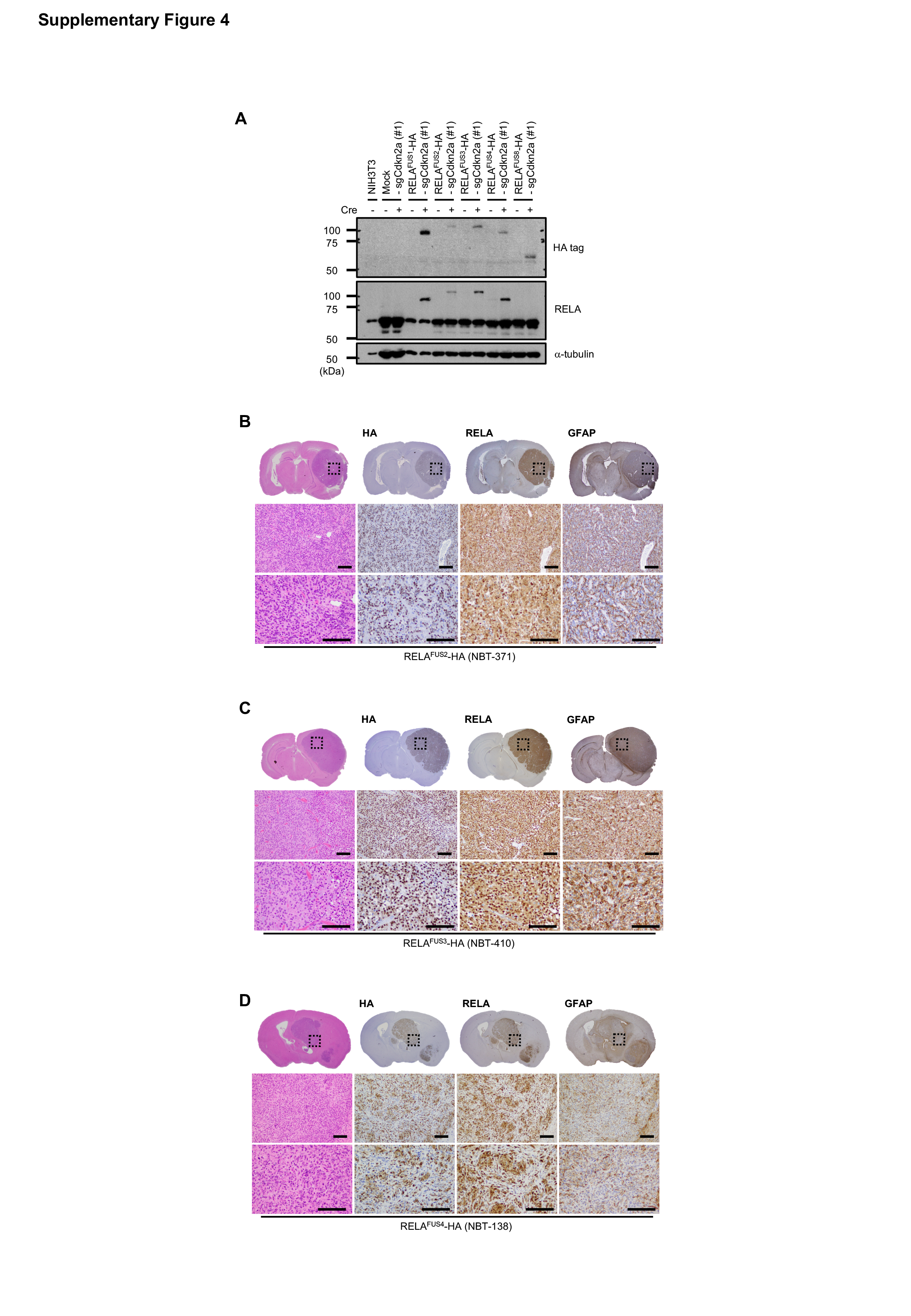

Supplement: Supplementary file 9 — Additional file 9: Figure S4. C11orf95-RELA fusion variants present diverse oncogenic potential (A) Western blot analysis for functional validation of pTomo-RELAFUS-HA-sgCdkn2a (#1) vector expression. NIH3T3 cells were infected with various pTomo lentiviruses as described. Subsequently, pCAG-Cre vector was transiently transfected to induce Cre-meditated recombination in the cells. Cell lysates were then subjected to immunoblot analysis with the indicated antibodies. Upper and lower bands in the RELA antibody detection indicate RELAFUS-HA protein and endogenous Rela protein, respectively. (B-D) Representative H&E and IHC analyses for HA tag, RELA and GFAP antibodies of the RELAFUS2-HA (B), RELAFUS3-HA (C) or RELAFUS4-HA (D) + sgCdkn2a (#1)-induced brain tumors in Nestin-Cre+/-;CAG-Cas9+/+ mice. Dashed boxes in the top panels denote the enlarged regions as shown in the bottom panels. Scale bars, 100 μm. [file 40478_2020_1080_MOESM9_ESM.tif]

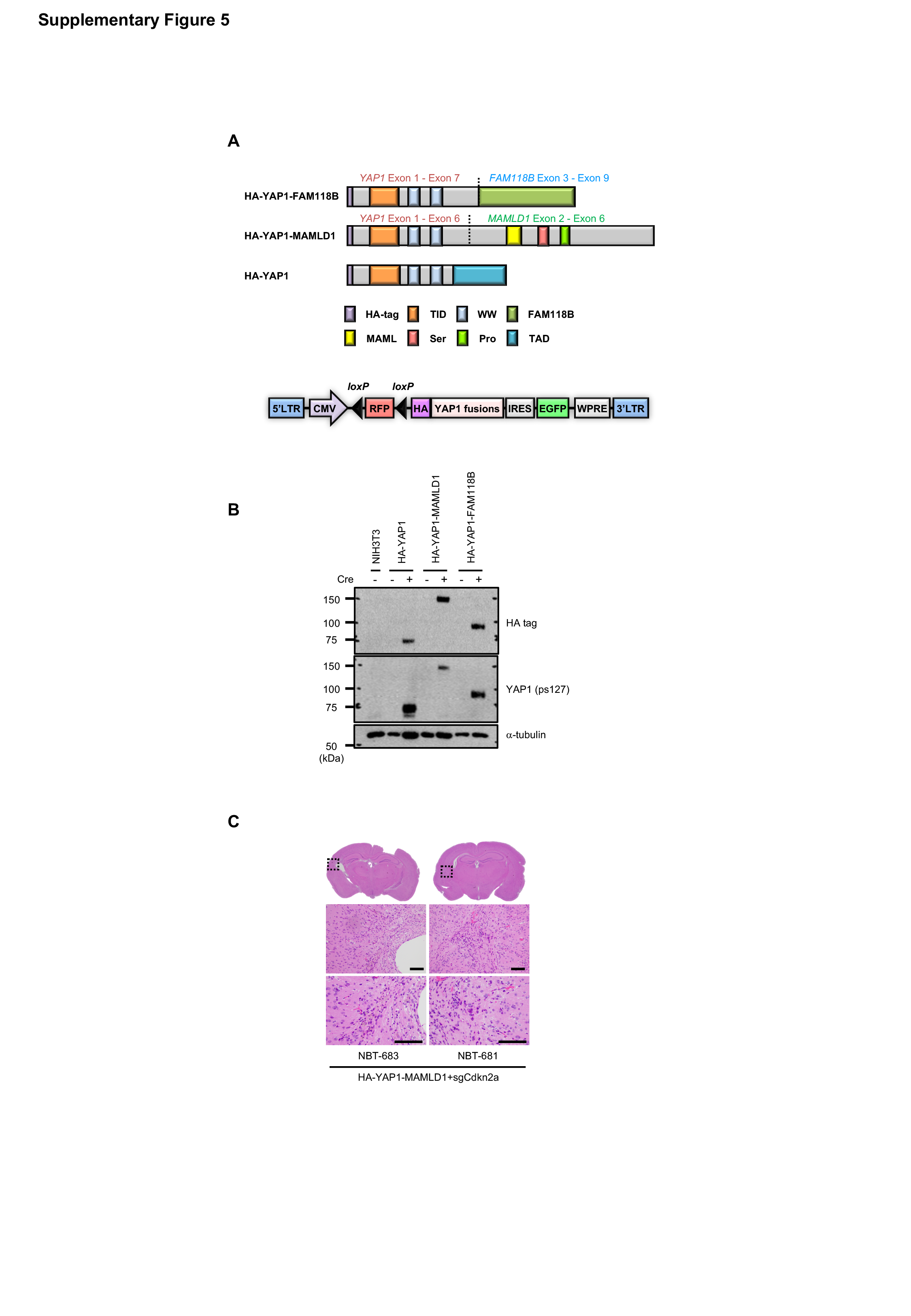

Supplement: Supplementary file 10 — Additional file 10: Figure S5. YAP1 fusions induces brain tumors with different morphological and histologic features from RELAFUS-induced tumors in mice. (A) Schematic presentation of the predicted protein products of YAP1 fusions and wild-type YAP1 with HA tag in the N-terminus in the pTomo-lentiviral vectors. TID, TEA domain-containing factor-interaction domain for TEAD binding; WW, protein–protein interaction domain; MAML, mastermind-like domain; Ser, serine-rich region; Pro, proline-rich region; TAD, transcriptional activation domain for TEAD. (B) Western blot analysis for pTomo-HA-YAP1 fusion vector expression. NIH3T3 cells were infected with various pTomo lentiviruses as described. Subsequently, pCAG-Cre vector was transiently transfected to induce Cre-meditated recombination in the cells. Cell lysates were then subjected to immunoblot analysis with the indicated antibodies. (C) Representative H&E images of the HA-YAP1-MAMLD1 + sgCdkn2a (#1)-induced brain tumors in Nestin-Cre+/-;CAG-Cas9+/+ mice. Dashed boxes in the top panels denote the enlarged regions as shown in the bottom panels. Scale bars, 100 μm. [file 40478_2020_1080_MOESM10_ESM.tif]
